# Hypoxic Preconditioning Restores NK Cell Metabolic Fitness by Attenuating MAPK Signaling to Potentiate Antitumor Immunity in Lung Cancer

**DOI:** 10.1002/advs.78759

**Published:** 2026-11-09

**Authors:** Dian Zhuang, Xiaoyue Zhu, Na Wang, Qianqian Liu, Lihua Chen, Yanzhuo Ye, Lingrui Shang, Shasha Huang, Cong Ma, Wei Kevin Zhang, Tao Xu, Jiaqi Sun, Huisheng Liu

**Affiliations:** ^1^ School of Biomedical Engineering Guangzhou Medical University Guangzhou Guangdong China; ^2^ Guangzhou National Laboratory Guangzhou Guangdong China; ^3^ College of Life Science and Technology Huazhong University of Science and Technology Wuhan Hubei China; ^4^ Institute of Cytology and Genetics School of Basic Medical Sciences Hengyang Medical School University of South China Hengyang Hunan China; ^5^ School of Forestry and Biotechnology Zhejiang A&F University Hangzhou China; ^6^ National Key Laboratory for Development and Utilization of Forest Food Resources Zhejiang A&F University Hangzhou China

**Keywords:** hypoxic preconditioning, MAPK signaling, mitochondrial metabolism, natural killer cells, solid tumor immunotherapy

## Abstract

Solid tumor hypoxia is a major barrier to NK cell‐based immunotherapy. To address this challenge, we establish a hypoxic preconditioning (HPC) model. We find that acute hypoxia (AH; 1% O_2_) significantly impairs NK92 cell cytotoxicity—such as granzyme B secretion and infiltration into lung cancer cell spheroids. In contrast, HPC pretreatment (gradual reduction from 5% to 1% O_2_) remarkably restores these functions. Mechanistically, transcriptomic analysis reveals that HPC markedly attenuates hypoxia ‐induced aberrant MAPK pathway activation. MAPK attenuation under normoxia recapitulates HPC ‐like protection by promoting mitochondrial biogenesis and metabolic remodeling, thereby enhancing the antitumor activity of NK92 cells both in vitro and in vivo. Notably, systemic MAPK inhibitor injection increases NKp46 expression on endogenous NK cells in BALB/c mice. Importantly, this strategy is also effective in human induced pluripotent stem cell (hiPSC)‐derived induced NK (iNK) cells, supporting broader evaluation across NK cell platforms. Collectively, this study identifies the MAPK–mitochondrial metabolic axis as a critical regulator of hypoxia‐induced NK cell dysfunction and proposes metabolic preconditioning as a strategy to overcome solid tumor hypoxia.

## Graphical abstract

Solid tumor hypoxia severely compromises natural killer cell function. This study demonstrates that hypoxic preconditioning preserves NK cell antitumor activity by limiting excessive stress ‐MAPK signaling and promoting mitochondrial metabolic fitness. Pharmacological MAPK attenuation reproduces this protective phenotype in NK92 and iPSC ‐derived NK cells, identifying the MAPK‒mitochondrial axis as a potential strategy for improving NK cell therapy in hypoxic tumors.

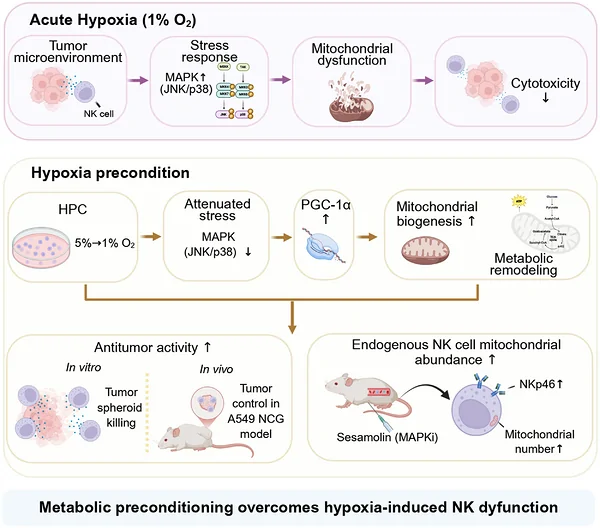

## 1 Introduction

Natural killer (NK) cells are key innate immune effectors capable of rapid, antigen‐independent cytotoxicity against malignant cells, making them promising candidates for cancer immunotherapy [1]. Adoptive NK cell therapies—including peripheral blood‐derived NK cells, NK92 cell lines, and stem cell‐derived induced NK (iNK) cells—have shown initial efficacy across tumor types [2]. Although promising, NK cell therapy still faces the critical challenge of the tumor microenvironment (TME) in clinical application [3], particularly chronic hypoxia [4].

Previous studies have firmly established that the hypoxic TME of solid tumors potently suppresses NK cell cytotoxicity and proinflammatory cytokine production [5]. In solid tumors, persistent hypoxia directly challenges this bioenergetic program by impairing mitochondrial respiration, reducing metabolic reserve, and disrupting redox homeostasis, thereby compromising perforin‐mediated cytotoxicity and cytokine production [6]. NK cells are highly dependent on metabolic fitness to sustain effector maturation, immune synapse formation, granule polarization, degranulation, and antibody‐dependent cellular cytotoxicity (ADCC) [7]. Emerging evidence further indicates that upon infiltration into tumors, NK cells undergo rapid, hypoxia‐driven transcriptional reprogramming—leading to swift functional exhaustion and loss of effector competence [8, 9]. Current NK cell engineering focuses mainly on enhancing tumor recognition (e.g., via CARs or affinity‐optimized receptors) [10, 11], neglecting the metabolic support required for durable effector function. Although hypoxia‐inducible factor‐1α (HIF‐1α) is widely recognized as a canonical hypoxia‐responsive factor [8], accumulating evidence indicates that hypoxia‐induced NK cell dysfunction cannot be fully explained by HIF‐1α‐dependent mechanisms [12, 13]. The key regulatory mechanisms underlying hypoxia‐induced metabolic collapse remain unclear, and effective metabolic intervention strategies are still lacking in clinical practice.

Hypoxic preconditioning (HPC) has been widely applied and has demonstrated a remarkable ability to enhance cellular resistance to hypoxia and ischemic stress. For example, HPC enhances mesenchymal stromal cell (MSC) survival, pro‐angiogenic factor expression, and reparative efficacy in ischemic myocardium [14, 15]; while clinically, hypoxic–hyperoxic preconditioning has been explored to reduce perioperative myocardial and endothelial injury and postoperative complications in coronary artery bypass surgery [16]. Whether a similar strategy can be applied to NK cells, however, remains unresolved. MAPK signaling comprises ERKs, SAPK/JNKs, and p38 MAPKs. Previous studies have shown that mitochondrial complex III‒derived ROS can participate in hypoxia‐induced activation of p38 MAPK, suggesting that MAPK signaling may function as a redox‐sensitive stress transduction module linking hypoxic stimulation to cellular adaptive responses [17, 18]. However, the role of MAPK signaling in NK cell responses to hypoxic stress—and whether it can serve as an intervention point for modulating NK cell adaptation to hypoxia—remains poorly investigated.

Here, we establish HPC as an ex vivo strategy to turn hypoxia from a barrier into a conditioning signal for NK cells. By gradually reducing oxygen tension from 5% to 1% O_2_, HPC enabled NK cells to preserve mitochondrial fitness and antitumor function during subsequent exposure to severe hypoxia. Integrated transcriptomic and functional analyses identified a p38/JNK‐centered MAPK–mitochondrial checkpoint as a central stress node linking hypoxic signaling to mitochondrial injury and NK cell dysfunction. Pharmacological attenuation of MAPK signaling under normoxia recapitulated key features of HPC by promoting PGC‐1α‒associated mitochondrial remodeling and metabolic reserve, thereby sustaining NK cell function under hypoxia. Importantly, these effects translated into enhanced tumor control and sustained NK cell persistence in lung cancer xenograft models and were further validated in human induced pluripotent stem cell (hiPSC) ‐derived NK cells—supporting the potential applicability of this strategy across distinct NK cell platforms. Together, our findings reframe hypoxia as a programmable conditioning cue and define MAPK‐directed metabolic reprogramming as a strategy to enhance NK cell environmental fitness for solid‐tumor immunotherapy.

## 2 Results

### 2.1 Hypoxia in the Tumor Microenvironment Suppresses the Proliferation and Cytotoxic Activity of NK92 Cells

To characterize the functional status of NK cells in the microenvironment of non‐small cell lung cancer (NSCLC) tissues, we first performed an analysis of the public single‐cell transcriptomic dataset [19]. This dataset includes immune cell profiles from patient tumor tissues, adjacent normal tissues, and mediastinal lymph nodes (mLNs). Based on canonical NK cell marker genes—including *KLRB1*, *NCR1*, and *NCAM1* [20, 21]—NK cell populations in tumor and adjacent normal tissues were identified and annotated. Subsequently, guided by key functional requirements for NK cell antitumor activity in the tumor microenvironment, NK cells were reclustered based on four hypothesis‐driven modules: *HIF‐1α*‐associated hypoxic response [22], *CXCR2*‐mediated chemotaxis and spatial infiltration, *CD69*‐associated activation and tissue residency, and *IL2RB*‐related cytokine responsiveness and survival potential [23]. Relative to NK cells from normal tissues, tumor‐infiltrating NK cells showed an increased proportion of the HIF‐1α‐associated hypoxic‐response cluster (Figure 1a). Further comparison of the transcriptional profiles of function‐associated genes showed that, compared with other clusters, the *HIF‐1α‒*associated cluster exhibited significantly reduced expression of cytotoxic effector molecules, including *GZMA*, *GZMB*, and *PRF1* [8]. In parallel, the expression of glycolysis‐related genes, including *GAPDH*, *LDHA*, and *PGK1* [24], was upregulated (Figure 1b). To assess the clinical relevance of HIF‐1α signaling, survival analysis was performed using the GSE72094 lung adenocarcinoma cohort. Among 398 patients with available survival information, higher HIF‐1α expression showed a trend toward poorer overall survival in the median‐based Kaplan‒Meier analysis (log‐rank *p* = 0.073; Figure S1a). When analyzed as a continuous variable, HIF‐1α expression was significantly associated with increased mortality in univariable Cox regression analysis (Figure S1b).

**FIGURE 1:**
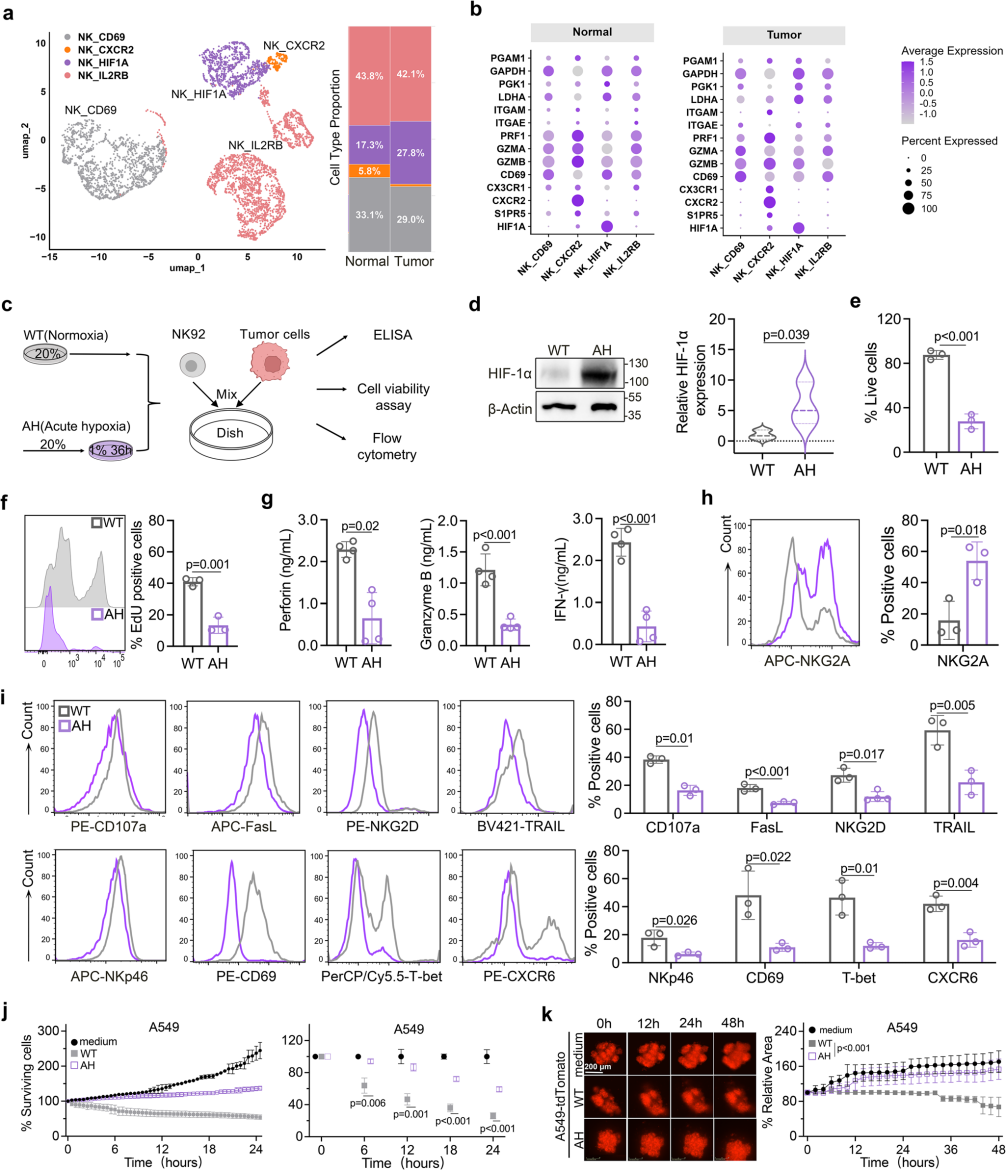
Hypoxia in the tumor microenvironment drives NK cell dysfunction and compromises antitumor immunity in lung cancer. (a) Single‐cell RNA sequencing (scRNA‐seq) data clustering analysis. Left panel: umap_1/umap_2 plot showing cell subcluster distribution. Right panel: Bar plot depicting the proportion of each subcluster across different sample groups. Data are derived from GSE198099. (b) Transcriptional expression levels of NK cell function‒associated factors in adjacent normal tissues and lung cancer tissues. (c) Schematic illustration of the experimental workflow for wild‐type (WT)‐ and acute hypoxia (AH)‐treated NK92 cells. Cells from the WT and AH groups were co‐cultured with tumor cells. (d) Western blot analysis of HIF‐1α protein expression levels in NK92 cells after AH treatment (*n* = 3). (e) Viability of NK92 cells following AH treatment was assessed by trypan blue exclusion assay and compared with that of WT cells (n = 3). (f) Proliferation of NK92 cells evaluated by EdU incorporation assay coupled with flow cytometry (n = 3). (g) Secretion of perforin‐1, granzyme B, and IFN‐γ in the supernatant of NK92 cells co‐cultured with A549 cells was measured by ELISA. (Effector‐to‐target [E:T] ratio = 4:1; co‐culture time = 4 h; *n* = 4). (h) Expression of the immune inhibitory receptor NKG2A on NK92 cells co‐cultured with A549 cells was analyzed by flow cytometry, with corresponding quantification (E:T = 4:1; time = 4 h; *n* = 3). (i) Expression of functional molecules in NK92 cells following stimulation with A549 cells was analyzed by flow cytometry, with corresponding quantification (E:T = 4:1; time = 4 h; *n* = 3). (j) Cytotoxic activity of WT‐ and AH‐treated NK92 cells against lung cancer cell lines A549‐tdTomato‐Luciferase, evaluated using the IncuCyte real‐time imaging system (E:T = 4:1; *n* = 3). (k) Cytotoxic activity of NK92 cells against A549‐tdTomato‐Luciferase 3D tumor spheroids, evaluated using the IncuCyte real‐time imaging system (E:T = 8:1; *n* = 3). Left panel: Representative red fluorescence images at indicated time points; scale bar = 200 µm. Right panel: Corresponding quantitative analysis. Data are presented as mean ± SD. Statistical significance in panel (d), (e), (f), (g), (h), (i), and (j) was determined using an unpaired two‐tailed Student's *t*‐test, except for panel (k), which was analyzed by two‐way ANOVA followed by multiple‐comparison tests.

To further mimic the hypoxic stress experienced by NK cells after entering the solid tumor microenvironment, NK92 cells were exposed to acute hypoxia (AH; 1% O_2_) in vitro (Figure 1c). Western blot analysis showed that AH treatment markedly promoted the accumulation of HIF‐1α protein, indicating effective activation of the hypoxic response (Figure 1d), while Trypan blue exclusion and EdU flow cytometry showed significantly reduced cell viability and S‐phase entry under AH (Figure 1e,f). These results indicated that acute hypoxia exerts a pronounced inhibitory effect on the expansion and maintenance of NK92 cells. Following co‐culture with A549 cells, AH‐treated NK92 cells secreted lower levels of perforin‐1, granzyme B, and the pro‐inflammatory cytokine IFN‐γ (Figure 1g). NKG2A is an important immune checkpoint receptor that transduces inhibitory signals through its intracellular ITIM motif [25]. After stimulation with A549 tumor cells, AH‐treated NK92 cells exhibited significantly increased expression of NKG2A (Figure 1h), suggesting that hypoxia may induce an immunosuppressive phenotype. In contrast, NKG2D, CD107a, FasL, and TRAIL, which are involved in target recognition, degranulation, and induction of target‐cell apoptosis [26], were significantly decreased. NKp46, CD69, T‐bet, and CXCR6, which are associated with NK cell activation, effector regulation, and tissue infiltration [27], were also markedly downregulated (Figure 1i).

To validate the effect of hypoxia on NK92 cell cytotoxicity from multiple perspectives, we combined a luciferase reporter system with IncuCyte real‐time live‐cell imaging using A549 and PC‐9 lung adenocarcinoma models [28] and H1299 p53‐null metastatic NSCLC model [29]. In all three target cell models, the killing efficiency of AH‐treated NK92 cells was significantly lower than that of normoxia control cells (Figure 1j; Figure S1c,e). To further model the 3D structural barrier of solid tumors, we established 3D tumor spheroid models using A549, PC‐9, and H1299 cells. AH treatment also significantly reduced the ability of NK92 cells to infiltrate into the core region of tumor spheroids (Figure 1k; Figure S1d). Taken together, these findings demonstrate that acute hypoxia not only inhibits the proliferation and survival of NK92 cells but also markedly reduces their antitumor immune activity by upregulating inhibitory receptors, downregulating cytotoxic effector molecules, and impairing tumor infiltration capacity.

### 2.2 Optimization of HPC Identifies HPC‐III as the Most Effective Regimen

Given that AH markedly impaired NK cell proliferation and cytotoxicity, we introduced an in vitro hypoxic preconditioning (HPC) strategy as a controllable stress‐buffering approach to enhance NK cell resilience against pathological hypoxic injury. To optimize the preconditioning parameters, three graded HPC regimens were designed and compared with normoxic control (WT) and AH groups. HPC‐I consisted of culture under 5% O_2_ for 2 d; HPC‐II involved sequential culture under 5% and 3% O_2_ for 2 d each; and HPC‐III employed progressive acclimation to 5%, 3%, and 1% O_2_ conditions. After preconditioning, cells were returned to normoxia and subsequently challenged with acute hypoxia to assess cytotoxic function (Figure S2a). As the preconditioning gradient increased from HPC‐I to HPC‐III, NK cells showed a stepwise improvement in hypoxia tolerance, as reflected by increased cell viability and restored expression of the activating receptor NKG2D (Figure S2b,c). Functional assays further showed that HPC significantly rescued NK cell cytotoxicity under acute hypoxia, with HPC‐III exhibiting the strongest protective effect (Figure S2d). Given that HPC‐III most effectively restored NK92 cell function, it was selected for subsequent experiments and is hereafter referred to as HPC (Figure 2a).

**FIGURE 2:**
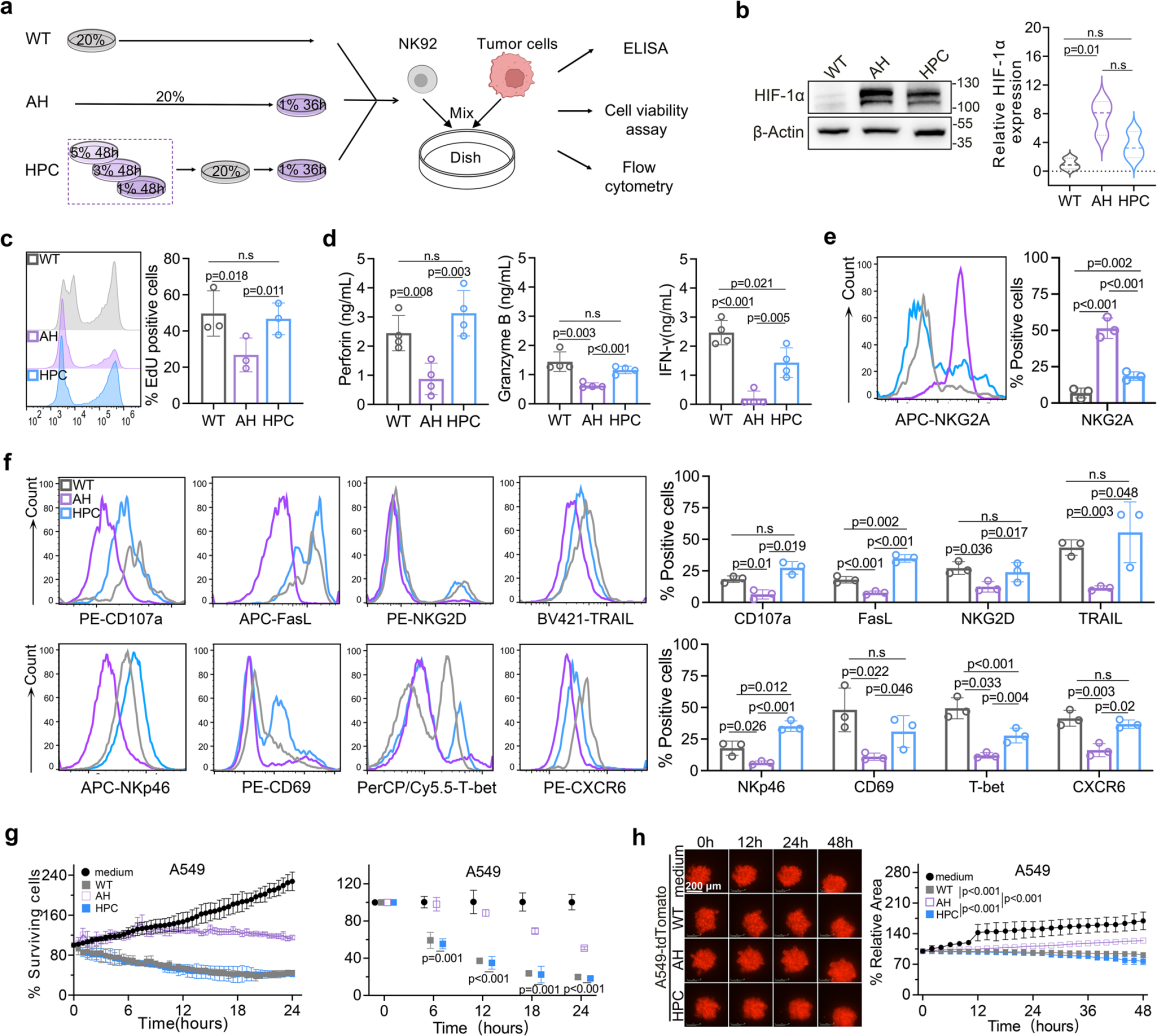
Hypoxia preconditioning restores NK92 cell cytotoxicity under acute hypoxia. (a) Schematic illustration of the experimental workflow. NK92 cells were first subjected to HPC (purple box), then returned to normoxic conditions, and subsequently exposed to 1% O_2_ for 36 h—simultaneously with the AH group. HPC in this and all subsequent figures represents the optimized HPC‐III conditioning protocol. Cells from the WT, AH, and HPC groups were co‐cultured with tumor cells. (b) Western blot analysis of HIF‐1α protein expression in NK92 cells (*n* = 3). (c) Proliferation of NK92 cells was evaluated by EdU incorporation assay coupled with flow cytometry (*n* = 3). (d) Secretion of perforin‐1, granzyme B, and IFN‐γ in the supernatant of NK92 cells co‐cultured with A549 cells was quantified by ELISA (E:T = 4:1; time = 4 h; *n* = 4). (e) Expression of the immune inhibitory receptor NKG2A on NK92 cells co‐cultured with A549 cells was analyzed by flow cytometry, with quantification (E:T = 4:1; time = 4 h; *n* = 3). (f) Expression of functional molecules in NK92 cells following stimulation with A549 cells was analyzed by flow cytometry, with corresponding quantification (E:T = 4:1; time = 4 h; *n* = 3). (g) Cytotoxic activity of WT‐, AH‐, and HPC‐treated NK92 cells against A549‐tdTomato‐Luciferase, evaluated using the IncuCyte real‐time imaging system (E:T = 4:1; *n* = 3). (h) Cytotoxic activity of NK92 cells against A549‐tdTomato‐Luciferase 3D tumor spheroids, evaluated using the IncuCyte real‐time imaging system (E:T = 8:1; *n* = 3). Left panel: Representative red fluorescence images at indicated time points; scale bar = 200 µm. Right panel: Corresponding quantitative analysis. Data are presented as mean ± SD. Statistical analyses were performed using IBM SPSS Statistics, except for panel (h), which was performed using GraphPad Prism. Statistical significance in panel (b), (c), (d), (e), (f), and (g) was determined using one‐way ANOVA followed by multiple‐comparison tests. Statistical significance in panel (h), which was analyzed by two‐way ANOVA followed by multiple‐comparison tests. n.s., not significant (*p*>0.05).

### 2.3 HPC Preserves Cytotoxic Function and Mitochondrial Fitness under Hypoxia

We next investigated whether HPC could reverse AH‐induced phenotypic impairment in NK92 cells. Compared with AH, HPC induced moderate HIF‐1α accumulation without excessive activation (Figure 2b) and significantly restored NK92 cell proliferation to near‐normoxic levels (Figure 2c). HPC also increased the secretion of perforin‐1, granzyme B, and IFN‐γ (Figure 2d), downregulated the inhibitory receptor NKG2A (Figure 2e), and restored the expression of activating receptors and effector molecules—including NKG2D, NKp46, TRAIL, FasL, and CD107a—as well as molecules associated with infiltration and functional activation—including CXCR6, T‐bet, and CD69 (Figure 2f). Consistent with this restored functional state, HPC‐treated NK92 cells exhibited markedly stronger cytotoxicity against tumor cells and improved infiltration into 3D tumor spheroids, accompanied by increased tumor cell apoptosis (Figure 2g,h; Figure S2e,f).

We next examined whether HPC‐mediated functional recovery was associated with mitochondrial metabolic remodeling. AH markedly increased mitochondrial reactive oxygen species (mtROS), reduced ATP production, and decreased mitochondrial area, whereas HPC largely reversed these defects (Figure 3a–c). Seahorse XF analysis showed that AH suppressed basal respiration and FCCP‐induced maximal respiration, while HPC restored oxygen consumption rate (OCR), spare respiratory capacity, and ATP‐linked respiration (Figure 3d,e). Extracellular acidification rate (ECAR) analysis further indicated improved glycolytic compensation in HPC‐treated cells under metabolic stress; in contrast, acute hypoxia impaired both mitochondrial respiration and compensatory glycolysis, suggesting a global bioenergetic defect in NK cells (Figure 3f,g). RNA sequencing (RNA‐seq) analysis of WT, AH, and HPC cells showed that 778 differentially expressed genes overlapped between the HPC versus AH and AH versus WT comparisons, whereas only 176 genes differed between WT and HPC groups, indicating that HPC partially reversed AH‐induced transcriptional changes (Figure 3h; Figure S3a). Consistently, AH downregulated chemotaxis‐ and cytotoxicity‐related genes—including *CCR5*, *CXCR1*, *CXCR6*, *CX3CR1*, *GZMA*, *CD53*, and *ITGA1*—whereas HPC restored their expression. HPC further increased the expression of antioxidant and redox‐related genes, including *GCLM*, *GSR*, *GSTO1*, *NQO1*, *PRDX1*, *PRDX3*, *SOD1*, *SOD2*, and *TXNRD1* (Figure S3b). Pathway analysis further revealed down‐regulation of the HIF‐1α signaling pathway and recovery of DNA replication, oxidative phosphorylation, mitochondrial function, and anti‐apoptotic pathways after HPC treatment (Figure 3i). In addition, HPC reduced AH‐induced upregulation of immunosuppressive receptors—including TGF‐β receptors, adenosine receptors, and immune checkpoint molecules [30, 31, 32] (Figure 3j)—suggesting reduced susceptibility to suppressive signals in the tumor microenvironment. Collectively, these results demonstrate that HPC reprograms NK cells to convert hypoxic stress into an adaptive metabolic state that sustains effector function under oxygen deprivation.

**FIGURE 3:**
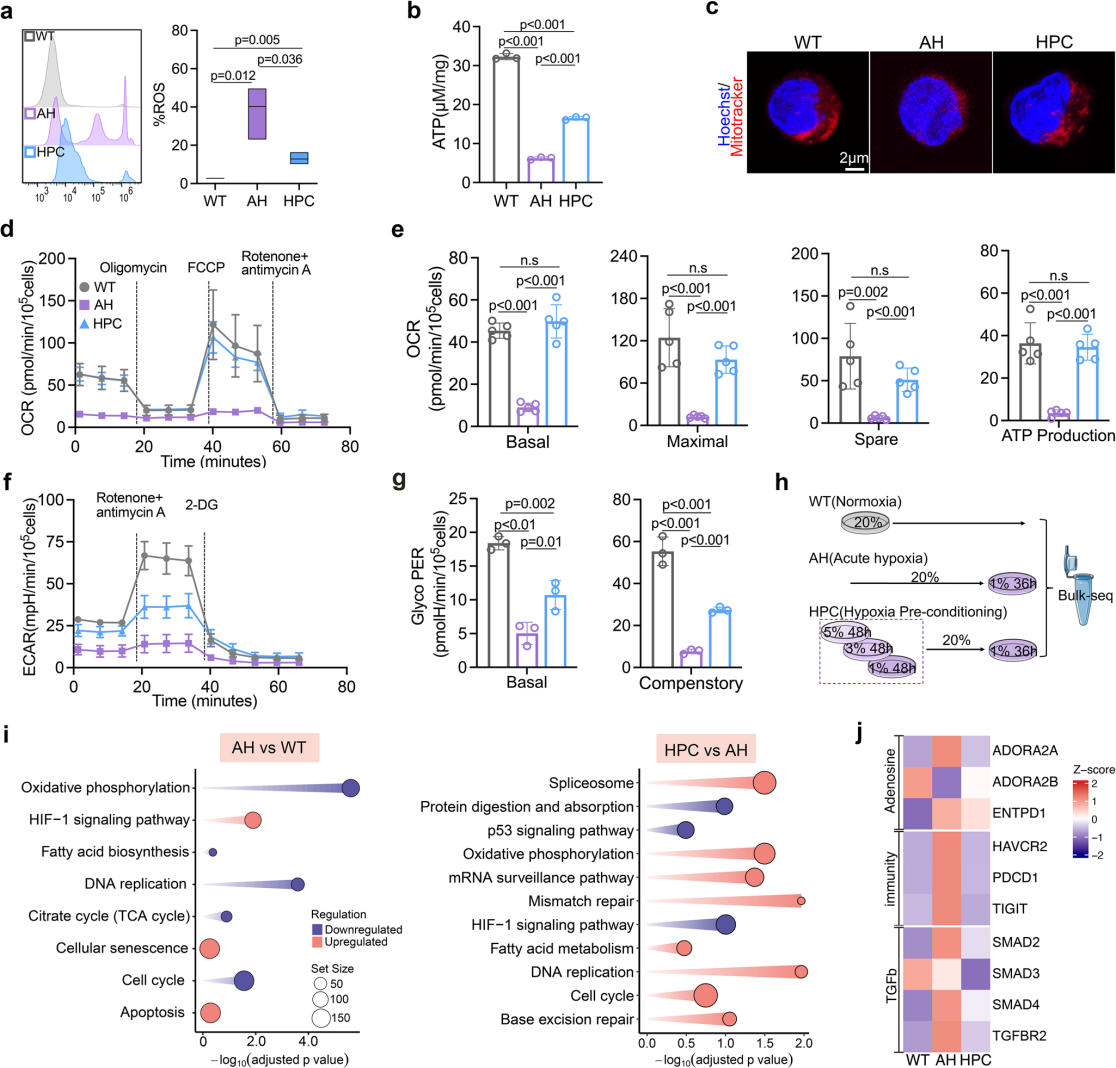
Hypoxia preconditioning restores mitochondrial function and bioenergetic fitness in NK92 cells. (a) Mitochondrial reactive oxygen species (mtROS) levels were assessed via flow cytometry coupled with the MitoSOX fluorescent probe, and results are presented as the percentage of mtROS‐positive cells (*n* = 3). (b) Intracellular ATP content was quantified using a fluorometric assay to evaluate cellular energy metabolic status (*n* = 3). (c) Live‐cell real‐time imaging was performed following MitoTracker staining to visualize and characterize mitochondrial morphology and network architecture; scale bar = 2 µm. MitoTracker Deep Red (red fluorescence) and Hoechst (blue fluorescence) were used to label mitochondria and nuclei, respectively (*n* = 10). (d) OCR analysis of WT‐, AH‐, and HPC‐treated NK92 cells using a Seahorse XF analyzer. Cells were seeded at 1 × 10^5^ cells per well and sequentially treated with oligomycin (1.5 µm), FCCP (1 µm), and rotenone/antimycin A (Rot/AA) (0.5 µm) to assess mitochondrial oxidative phosphorylation (*n* = 5). (e) Quantitative analysis of basal respiration, maximal respiration, spare respiratory capacity, and ATP production derived from OCR profiles (*n* = 5). (f) ECAR was measured in WT‐, AH‐, and HPC‐treated NK92 cells to evaluate aerobic glycolysis following the sequential addition of Rot/AA and 2‐deoxy‐D‐glucose (2‐DG) (*n* = 3). (g) Quantification of basal glycolysis and compensatory glycolysis (the glycolytic response following mitochondrial inhibition) (*n* = 3). (h) Schematic illustration of the experimental workflow. NK92 cells were subjected to HPC (purple box), followed by reoxygenation, then co‐exposed to 1% O_2_ for 36 h alongside the AH group. Cells from the WT, AH, and HPC groups were subjected to bulk RNA‐seq analysis (*n* = 3). (i) The bubble plot of Kyoto Encyclopedia of Genes and Genomes (KEGG) enrichment analysis shows pathways associated with metabolic regulation and cell survival. Specifically, left panel displayed pathways changed in AH versus WT, whereas right panel depicted pathways changed in HPC versus AH (*n* = 3). (j) Heatmap of the predicted potential responses of NK cells to tumor microenvironment‐related inhibitory factors (including TGF‐β [TGFb] receptors, adenosine receptors, and other immunosuppressive receptors) based on transcriptional analysis. Data are presented as mean ± SD. Statistical analyses were performed using IBM SPSS Statistics. Statistical significance in panel (a), (b), (e), and (g) was analyzed by one‐way ANOVA followed by multiple‐comparison tests. n.s, not significant (*p* > 0.05).

### 2.4 HPC‐Associated Transcriptional Recovery Identifies Stress‐Activated p38/JNK Signaling as a Driver of Hypoxia‐Induced NK92 Cell Dysfunction

To define the molecular basis of HPC‐mediated recovery, we focused on pathways activated by AH but normalized after HPC treatment. MAPK signaling was prominently enriched under AH and reduced following HPC (Figure 4a). Across individual NK cell samples from the GSE131907 dataset, the hypoxia/HIF1A signature score strongly correlated with MAPK signaling activity but negatively correlated with NKG7 expression, while MAPK signaling exhibited a modest nonlinear association with NKG7 expression (Figure S4a), suggesting a potential link between hypoxic stress, MAPK signaling, and NK cell functional states. Analysis of the major MAPK branches further showed reduced p38‐, JNK‐, and ERK‐related transcriptional programs after HPC (Figure S4b). Consistently, MAPK signaling was enriched in tumor‐infiltrating NK cells from NSCLC samples compared with adjacent normal tissues (Figure 4b).

**FIGURE 4:**
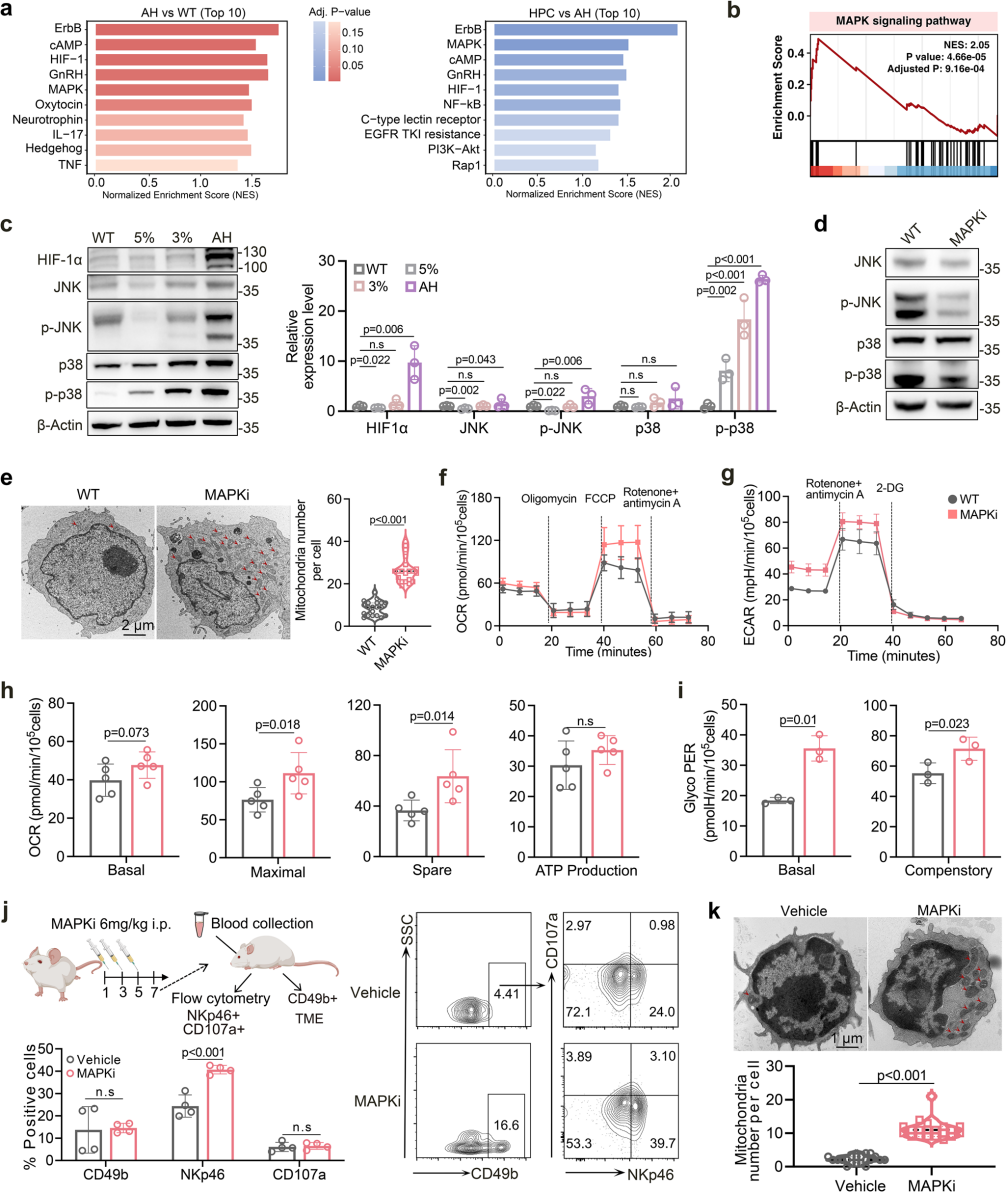
MAPK overactivation underlies the metabolic dysfunction induced by hypoxia in NK92 cells. (a) GSEA using KEGG pathway gene sets identified the top ten most significantly enriched signaling pathways among those upregulated in AH versus WT (left panel) and downregulated in HPC versus AH (right panel) (*n* = 3). (b) GSEA of NK cells isolated from tumor tissues versus adjacent normal tissues (Figure 1a) confirmed significant upregulation of the MAPK signaling pathway in tumor‐infiltrating NK cells (*n* = 3). (c) Western blot analysis revealed an oxygen‐dependent activation of the p38 and JNK pathways in NK92 cells. Graded hypoxia (20%, 5%, 3%, and 1% O_2_) for 18 h triggered a stepwise increase in the phosphorylation of p38 (Thr180/Tyr182) and JNK (Thr183/Tyr185) (left), with significant upregulation confirmed by densitometric quantification (right; *n* = 3). (d) Pharmacological attenuation of MAPK with a MAPK inhibitor (sesamolin, 5 µM; normoxic pretreatment for 48 h) effectively suppressed p38 and JNK phosphorylation in NK92 cells (*n* = 3). (e) Transmission electron microscopy (TEM) images and quantitative analysis of mitochondrial number per NK92 cell in the MAPKi‐treated group versus the WT control group. The red arrow demarcates the predominant subcellular localization of mitochondria; scale bar = 2 µm (*n* = 20). (f) Oxygen consumption rate (OCR) was measured in WT‐and MAPKi‐treated NK92 cells at a density of 1 × 10^5^ per well using a Seahorse XF analyzer to assess oxidative phosphorylation levels (*n* = 5). (g) Extracellular acidification rate (ECAR) was measured in WT‐ and MAPKi‐treated NK92 cells to evaluate aerobic glycolysis following the sequential addition of rotenone/antimycin A (Rot/AA) and 2‐deoxy‐D‐glucose (2‐DG) (*n* = 3). (h) Quantitative analysis of basal respiration, maximal respiration, spare respiratory capacity, and ATP production derived from OCR profiles (*n* = 5). (i) Quantification of basal glycolysis and compensatory glycolysis derived from ECAR profiles (*n* = 3). (j) Top left: Schematic of the experimental timeline showing that BALB/c mice received three intraperitoneal (i.p.) injections of MAPK inhibitor or vehicle at 48 h intervals, followed by blood collection 48 h after the final injection. Bottom left: Statistical quantification of NKp46 expression and CD107a degranulation levels (*n* = 4). Right: Representative flow cytometry plots and quantification of CD49b^+^ NK cells and NKp46/CD107a expression within the CD49b^+^ population in peripheral blood PBMCs. (k) Representative transmission electron microscopy images of sorted peripheral blood CD49b^+^ NK cells from vehicle‐ and sesamolin‐treated mice, with mitochondria indicated by red arrowheads. Quantification of mitochondrial number per cell is shown (*n* = 20 cells). Data are presented as mean ± SD. Statistical analyses were performed using IBM SPSS Statistics. Statistical significance between two groups in panel (h), (i), (j), and (k) was determined using an unpaired two‐tailed Student's *t*‐test. Statistical significance in panel (c) was analyzed by one‐way ANOVA followed by multiple‐comparison tests. n.s, not significant (*p*>0.05).

Because p38 MAPK and JNK are the two major stress‐activated MAPK branches most closely associated with hypoxic and oxidative stress responses [33, 34], we next examined their phosphorylation status. Western blot analysis revealed oxygen tension‐dependent activation of both pathways. As oxygen levels decreased from 20% to 5%, 3%, and 1% O_2_ (AH), phosphorylation of p38 MAPK (Thr180/Tyr182) and JNK (Thr183/Tyr185) increased progressively, whereas ERK phosphorylation showed no substantial change (Figure 4c; Figure S4c). These data indicate that hypoxia activates a stress‐associated p38/JNK MAPK program in NK92 cells, rather than merely altering MAPK‐related transcriptional signatures.

To further examine the functional link between stress‐MAPK activation and mitochondrial dysfunction, we first assessed the major MAPK branches in WT, AH, and HPC‐treated NK92 cells. Western blot analysis showed that AH increased JNK and p38 phosphorylation, whereas these changes were attenuated after HPC; ERK phosphorylation showed no substantial change (Figure S4d). We then treated NK92 cells with the MAPK activator anisomycin under normoxic conditions. Anisomycin strongly induced JNK and p38 phosphorylation and concomitantly increased mtROS accumulation, partially recapitulating the mitochondrial stress phenotype observed under AH conditions (Figure S4e,f). Moreover, reactivation of MAPK signaling in HPC‐treated cells with anisomycin increased intracellular ROS under subsequent hypoxia and reduced GZMB expression, partially reversing the protective phenotype conferred by HPC (Figure S4j,k). Together, these findings support a model in which HPC enhances antioxidant and redox‐defense capacity, thereby limiting hypoxia‐induced ROS accumulation and excessive p38/JNK activation, while preventing the downstream mitochondrial stress and impairment of NK cell effector function.

### 2.5 Sesamolin‐Mediated MAPK Attenuation Promotes PGC‐1α‐Associated Mitochondrial Biogenesis and Metabolic Fitness in NK92 Cells

These gain‐of‐function results suggested that MAPK hyperactivation contributes to inducing mitochondrial stress, prompting us to test whether pharmacological MAPK attenuation could rescue NK92 cells from AH‐induced dysfunction. We used sesamolin (MAPKi), a natural lignan compound reported to exert antioxidant and immunomodulatory effects and to suppress MAPK phosphorylation cascades, as a MAPK‐modulating intervention [35, 36]. Under normoxic conditions, sesamolin treatment for 48 h markedly reduced basal p38 and JNK phosphorylation, while ERK phosphorylation showed no substantial change (Figure 4d; Figure S9b). In contrast to HIF‐1α inhibition with PX‐478 [37], which did not substantially restore mitochondrial function, sesamolin markedly reduced mtROS accumulation, indicating that excessive MAPK activation is a major contributor to AH‐induced mitochondrial dysfunction (Figure S4i,j). Furthermore, treatment with the mitochondrial ROS scavenger MitoTEMPO reduced intracellular ROS accumulation and p38 phosphorylation under AH conditions (Figure S4k), suggesting that excessive ROS production contributes to hypoxia‐induced p38 activation. To shorten the time required for HPC and enhance clinical translatability, we next asked whether pharmacological attenuation of MAPK signaling under normoxic conditions could recapitulate the metabolic‐priming effects of HPC. MAPKi pretreatment increased mitochondrial abundance and improved metabolic reserve in NK92 cells, as evidenced by transmission electron microscopy and Seahorse analysis (Figure 4e–i). Systemic sesamolin treatment did not significantly alter the frequency of CD49b^+^ NK cells or CD107a^+^ cells, but significantly increased the frequency of NKp46^+^ cells within the CD49b^+^ NK cell population (Figure 4j). Consistently, systemic sesamolin treatment increased mitochondrial number in sorted peripheral‐blood CD49b^+^ NK cells from BALB/c mice, extending this mitochondrial remodeling phenotype to endogenous NK cells in vivo (Figure 4k; Figure S5a).

To explore the transcriptional basis underlying MAPKi‐induced mitochondrial expansion and metabolic priming, we performed RNA‐seq analysis of WT and MAPKi‐treated NK92 cells under normoxic conditions (Figure S5b). MAPKi pretreatment upregulated mitochondrial biogenesis‐ and quality control‐related genes—including *PPARGC1A* (PGC‐1α), which was further confirmed by quantitative RT‐PCR (Figure S5c–e). Given the central role of PGC‐1α in mitochondrial biogenesis and oxidative metabolism [38], we next examined its functional contribution to the MAPKi‐induced metabolic phenotype. Knockdown of PGC‐1α diminished the MAPKi‐associated improvement in mitochondrial respiration, including basal and maximal respiration, spare respiratory capacity, and ATP‐linked respiration (Figure S5f–h). Together, these findings identify PGC‐1α as a functional mediator linking MAPK attenuation to enhanced mitochondrial biogenesis and respiratory fitness, with corresponding mitochondrial remodeling validated in endogenous NK cells in vivo.

### 2.6 Normoxic MAPK Attenuation Functionally Recapitulates HPC by Enhancing Mitochondrial Fitness and Protecting NK92 Cells against Hypoxic Stress

To test whether MAPK attenuation‐mediated bioenergetic priming could improve NK92 cell function under hypoxia, MAPKi‐pretreated cells were exposed to AH (shortened as i+AH) and compared with WT and AH groups (Figure 5a). MAPKi pretreatment significantly restored NK92 cell viability and proliferation under hypoxic challenge (Figure 5b,c), and increased the secretion of perforin‐1, granzyme B, and IFN‐γ (Figure 5d). It also reduced NKG2A expression while restoring key functional molecules, including NKG2D, NKp46, TRAIL, FasL, and CD107a (Figure 5e,f). Consistently, i + AH markedly enhanced NK92 cell cytotoxicity against A549, PC‐9, and H1299 cells in luciferase‐based assays and real‐time killing analysis, and improved antitumor activity in 3D tumor spheroid models (Figure 5g,h; Figure S6a–c).

**FIGURE 5:**
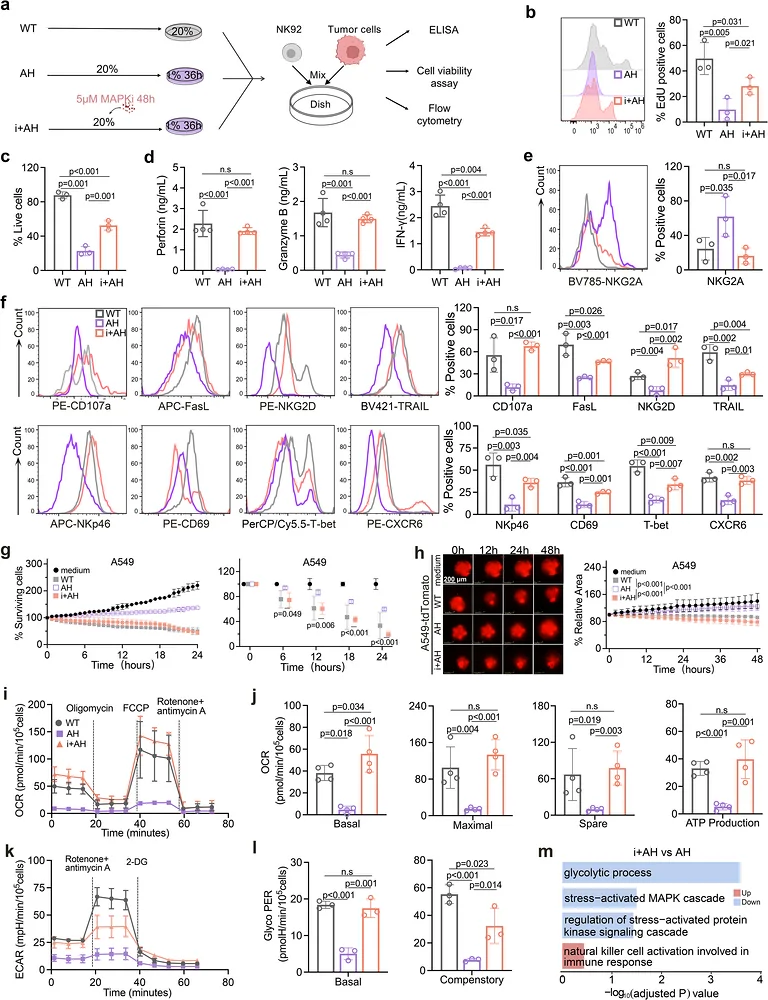
MAPK attenuation restores NK92 cell cytotoxicity and mitochondrial fitness under acute hypoxia. (a) Schematic illustration of the experimental workflow. NK92 cells were categorized into three groups: WT, AH, and i+AH. Cells in the i+AH group were pretreated with a MAPK inhibitor, sesamolin (5 µm), under normoxic conditions for 48 h. Thereafter, both AH and i+AH groups were exposed to hypoxia (1% O_2_) for 36 h. Following these treatments, NK92 cells from all groups were co‐cultured with tumor cells to evaluate their functional capacity. (b) Cell proliferation of NK92 cells under WT, AH, and i+AH treatment, assessed by EdU assay coupled with flow cytometry (*n* = 3). (c) Cell viability of NK92 cells following WT, AH, and i+AH treatment, determined by trypan blue exclusion assay (*n* = 3). (d) Secretion of perforin‐1, granzyme B, and IFN‐γ in the supernatant of NK92 cells co‐cultured with A549 cells was quantified by ELISA (E:T = 4:1; time = 4 h; *n* = 4). (e) Expression of the immune inhibitory receptor NKG2A on NK92 cells co‐cultured with A549 cells was analyzed by flow cytometry, with quantification (E:T = 4:1; time = 4 h; *n* = 3). (f) Expression of functional molecules in NK92 cells following stimulation with A549 cells was analyzed by flow cytometry, with corresponding quantification (E:T = 4:1; time = 4 h; *n* = 3). (g) Cytotoxic activity of WT‐, AH‐, and i+AH‐treated NK92 cells against A549‐tdTomato‐Luciferase, evaluated using the IncuCyte real‐time imaging system (E:T = 4:1; *n* = 3). (h) Cytotoxic activity of WT‐, AH‐, and i+AH‐treated NK92 cells against A549‐tdTomato‐Luciferase 3D tumor spheroids, evaluated using the IncuCyte real‐time imaging system; scale bar = 200 µm (E:T = 8:1; *n* = 3). Left panel: Representative red fluorescence images at indicated time points. Right panel: Corresponding quantitative analysis. (i) Oxygen consumption rate (OCR) was measured in WT‐, AH‐, and i+AH‐treated NK92 cells at a density of 1 × 10^5^ per well using a Seahorse XF analyzer to assess oxidative phosphorylation levels (*n* = 4). (j) Quantitative analysis of basal respiration, maximal respiration, spare respiratory capacity, and ATP production derived from OCR profiles (*n* = 4). (k) Extracellular acidification rate (ECAR) was measured in WT‐, AH‐, and i+AH‐treated NK92 cells to evaluate aerobic glycolysis following the sequential addition of Rot/AA and 2‐DG (*n* = 3). (l) Quantification of basal glycolysis and compensatory glycolysis (*n* = 3). (m) Differential pathway analysis of i+AH versus AH cells showed downregulation of stress‐activated MAPK‐related programs and enrichment of pathways associated with glycolytic regulation and NK cell activation (*n* = 3). Data are presented as mean ± SD. Statistical analyses were performed using IBM SPSS Statistics, except panel (h), which was performed using GraphPad Prism. Statistical significance in panel (b), (c), (d), (e), (f), (g), (j), and (l) was determined using one‐way ANOVA followed by multiple‐comparison tests. Statistical significance in panel (h), which was analyzed by two‐way ANOVA followed by multiple‐comparison tests. n.s, not significant (*p* > 0.05).

We next assessed mitochondrial function under hypoxia. Compared with AH alone, i+AH preserved mitochondrial area, reduced mtROS accumulation, and restored ATP production (Figure S6d–f). Notably, *PPARGC1A* knockdown partially reversed the MAPKi‐associated reduction in intracellular ROS under subsequent hypoxia (Figure S6g), further supporting a functional role for PGC‐1α in MAPKi‐mediated redox control. Seahorse analysis further showed improved OCR, basal and maximal respiration, ATP‐linked respiration, spare respiratory capacity, and compensatory glycolytic activity in MAPKi‐pretreated cells under hypoxia (Figure 5i–l). Consistently, combined *MAPK8/MAPK14* knockdown reduced hypoxia‐induced ROS and preserved mitochondrial respiration, including basal and maximal respiration, spare respiratory capacity, and ATP‐linked respiration (Figure S7d–g). These results indicate that MAPK attenuation preserves mitochondrial fitness and metabolic flexibility under hypoxic stress.

RNA‐seq analysis of WT, AH, and i+AH cells further showed that MAPKi pretreatment attenuated AH‐induced hypoxia response and MAPK signaling, while restoring pathways associated with mitochondrial metabolism, oxidative phosphorylation, and NK cell effector function (Figure 5m; Figure S7a). Venn diagram analysis showed that MAPKi pretreatment partially reversed the AH‐induced transcriptional program, including 27% of AH‐upregulated genes and 6.4% of AH‐downregulated genes. Rather than restoring a WT‐like state, MAPKi‐treated cells under AH adopted a distinct adaptive transcriptional profile (Figure S7b). Heatmap analysis confirmed recovery of chemotaxis‐, cytotoxicity‐, and activation‐related genes suppressed by AH, together with reversal‐like reprogramming of glycolysis, HIF‐1α signaling, and oxidative phosphorylation genes (Figure S7c). Together, these findings demonstrate that normoxic MAPK attenuation partially mimics HPC by priming mitochondrial metabolism and enhancing NK92 cell resistance to hypoxic stress.

### 2.7 Either HPC or MAPK Attenuation Enhances NK92 Cell Persistence and Antitumor Function In Vivo

To evaluate in vivo antitumor efficacy, we established a subcutaneous A549‐tdTomato‐Luciferase xenograft model and treated tumor‐bearing mice with PBS, WT‐, HPC‐, and MAPKi‐pretreated NK92 cells (Figure 6a). Bioluminescence imaging (BLI) and longitudinal tumor measurements showed that WT NK92 cells exerted a modest antitumor effect, whereas HPC‐ and MAPKi‐pretreated cells achieved stronger and more sustained tumor control, with the lowest tumor burden observed in the HPC group (Figure 6b–d). Both treatment groups also prolonged overall survival without overt body‐weight loss (Figure 6e,f). Flow cytometry performed 7 and 14 days after the final NK cell infusion further showed that HPC‐ and MAPKi‐pretreated NK92 cells displayed increased tumor infiltration and prolonged persistence in peripheral blood compared with WT cells (Figure 6g,h). Consistently, NK92 cells recovered from tumor‐bearing mice exhibited significantly increased p‐p38 positivity compared with those from healthy mice, whereas ex vivo sesamolin pretreatment markedly attenuated this increase (Figure S7h), further supporting tumor‐associated MAPK/p38 hyperactivation and its suppression by MAPK inhibition. Together, these findings indicate that HPC and MAPKi pretreatment enhance the in vivo persistence, tumor infiltration, and antitumor activity of NK92 cells, leading to durable suppression of tumor growth.

**FIGURE 6:**
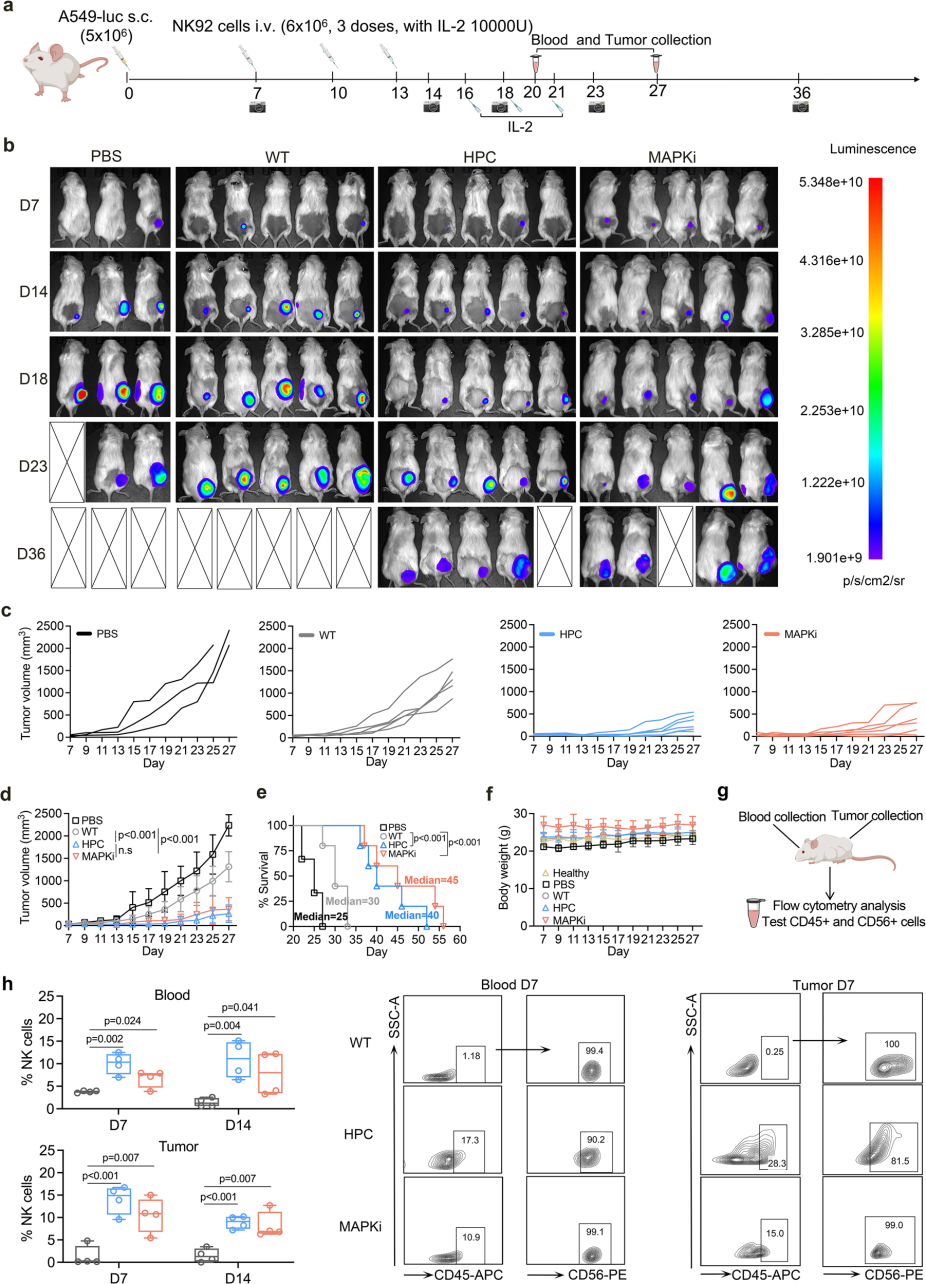
Either HPC or MAPK modulation improves in vivo antitumor efficacy and persistence of NK92 cells. (a) Schematic illustration of the in vivo experimental workflow. A549‐tdTomato‐Luciferase cells (5 × 10^6^ per mouse) were implanted subcutaneously into the dorsal flank of NSG mice to establish a human tumor xenograft model. On day 7 post‐implantation, mice were randomized into four groups and received intravenous administrations of PBS or 6 × 10^6^ NK92 cells (WT‐, HPC‐, or MAPKi‐pretreated), respectively. Each NK cell infusion was supplemented with 10 000 U of IL‐2 to support in vivo persistence. The treatment was administered every three days for a total of three injections. Tumor progression was monitored via bioluminescence imaging and manual caliper measurements. (b) Representative in vivo bioluminescence images of tumor burden in PBS‐, WT‐, HPC‐, and MAPKi‐treated mice (PBS: *n* = 3; WT/HPC/MAPKi: *n* = 5 per group). (c) Individual tumor growth trajectories across treatment groups. Tumor volume was quantified every two days using digital calipers; D‐luciferin (150 mg kg^−1^, i.p.) was administered prior to imaging (PBS: *n* = 3; WT/HPC/MAPKi: *n* = 6 per group). (d) Tumor growth kinetics. Tumor volumes were measured every 2 d (PBS: *n* = 3; WT/HPC/MAPKi: *n* = 6 per group). (e) Kaplan–Meier survival analysis comparing overall survival among treatment cohorts (PBS: *n* = 3; WT/HPC/MAPKi: *n* = 5 per group). (f) Longitudinal body weight monitoring. Mean body weight changes are shown as mean ± SD (Healthy control and PBS: *n* = 3; WT/HPC/MAPKi: *n* = 6 per group). (g) Experimental timeline for NK92 cell persistence analysis. NK92 cells were administered on the indicated treatment days, and peripheral blood and tumor tissues were collected 7 and 14 d after the thirdNK92 cell infusion. Human NK92 cells were identified as CD45^+^CD56^+^ cells by flow cytometry and quantified in peripheral blood and tumor tissues. (h) Quantification and representative flow cytometry plots showing NK92 cell frequencies in peripheral blood and tumor tissues on days 7 and 14 after the third NK cell infusion (WT/HPC/MAPKi: *n* = 3 per group). Data are presented as mean ± SD. Longitudinal tumor growth and body‐weight changes were analyzed using two‐way ANOVA followed by multiple‐comparison tests. For NK92 persistence analyses, comparisons among WT, HPC, and MAPKi groups at each indicated time point were performed using one‐way ANOVA followed by multiple‐comparison tests. Survival curves were compared using the log‐rank test. n.s, not significant (*p*>0.05).

### 2.8 MAPK Attenuation Enhances Hypoxia Tolerance in Both NK92 and iPSC‐Derived iNK Cells

To assess the broader applicability of MAPK attenuation in a clinically relevant NK cell platform, we established an iNK model using a hiPSC through embryoid body formation and CD34^+^ hematopoietic specification (Figure 7a). CD34^+^ progenitors remained above 50% on day 12 and the resulting iNK cells stably expressed CD45, CD56, and CD16, with minimal contamination by CD3^+^ T cells (Figure 7b; Figure S8a,b). Their functional receptor profile was highly comparable to that of NK92 cells (Figure S8c), confirming the reliability of this differentiation system. Under AH, iNK cells exhibited functional impairment similar to NK92 cells, whereas MAPKi pretreatment reduced mtROS and restored perforin‐1, granzyme B, and IFN‐γ secretion (Figure 7c,d). MAPKi pretreatment also upregulated multiple activating receptors and intracellular effector molecules in iNK cells (Figure 7e,f). In a parallel HPC experiment, HIF‐1α protein remained elevated under hypoxia but was moderately reduced by HPC pretreatment, consistent with the pattern observed in NK92 cells (Figure S8g).

**FIGURE 7:**
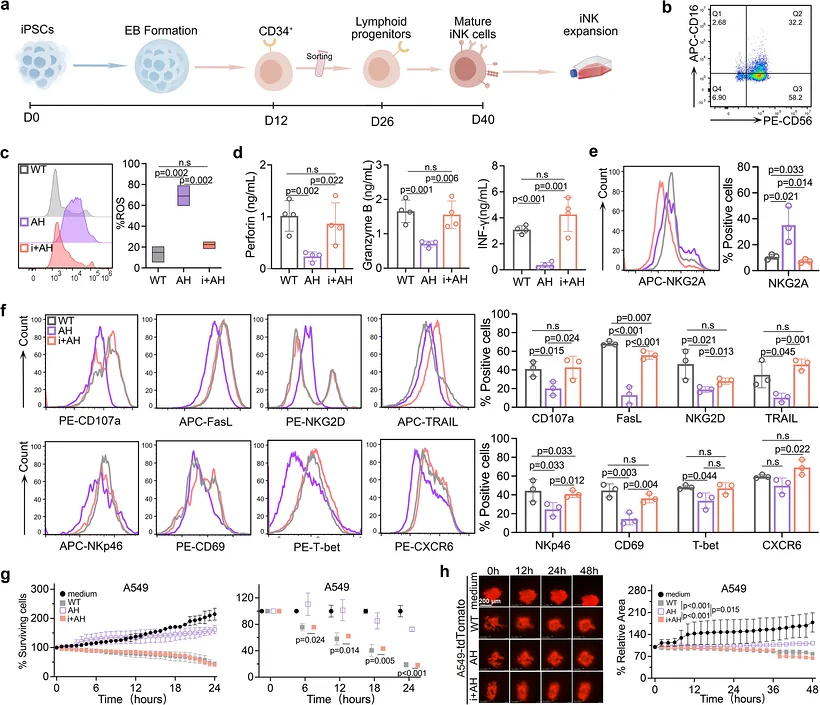
Conserved efficacy of MAPK attenuation in enhancing antitumor functions of both NK92 and iNK cells under hypoxia. (a) Schematic representation of the stepwise differentiation of induced natural killer (iNK) cells from induced human pluripotent stem cells (iPSCs). Differentiation was performed using a commercially available stem cell–to–NK cell differentiation kit, following a three‐stage protocol: (i) embryoid body (EB) formation; (ii) specification into CD34^+^ hematopoietic progenitor cells; and (iii) commitment to the lymphoid lineage and terminal maturation into functional iNK cells. Created with BioGDP.com. (b) Flow cytometry plots showing the expression of CD16 and CD56 on day 40 of iPSC differentiation. (c) Mitochondrial reactive oxygen species (mtROS) levels in iNK cells following i+AH treatment were quantified by flow cytometry using MitoSOX Red, with data expressed as the percentage of mtROS‐positive cells (*n* = 3). (d) Secretion of perforin‐1, granzyme B, and IFN‐γ in the supernatant of iNK cells co‐cultured with A549 cells was quantified by ELISA (E:T = 4:1; time = 4 h; *n* = 4). (e) Expression of the immune inhibitory receptor NKG2A on iNK cells co‐cultured with A549 cells was analyzed by flow cytometry, with quantification (E:T = 4:1; time = 4 h; *n* = 3). (f) Expression of functional molecules in iNK cells following stimulation with A549 cells was analyzed by flow cytometry, with quantification (E:T = 4:1; time = 4 h; *n* = 3). (g) Cytotoxic activity of WT‐, AH‐, and i+AH‐treated iNK cells against A549‐tdTomato‐Luciferase, evaluated using the IncuCyte real‐time imaging system (E:T = 4:1; *n* = 3). (h) Cytotoxic activity of WT‐, AH‐, and i+AH‐treated iNK cells against A549‐tdTomato‐Luciferase 3D tumor spheroids, evaluated using the IncuCyte real‐time imaging system; scale bar = 200 µm (E:T = 8:1; *n* = 3). Left panel: Representative red fluorescence images at indicated time points. Right panel: Corresponding quantitative analysis. Data are presented as mean ± SD. Statistical analyses were performed using IBM SPSS Statistics, except for panel (h), which was performed using GraphPad Prism. Statistical significance in panel (c), (d), (e), (f), and (g) was determined using one‐way ANOVA followed by multiple‐comparison tests. Statistical significance in panel (h), which was analyzed by two‐way ANOVA followed by multiple‐comparison tests. n.s, not significant (*p* > 0.05).

Luciferase‐based cytotoxicity assays demonstrated that MAPKi pretreatment enhanced sustained iNK cell killing of A549, PC‐9, and H1299 under AH, while 3D spheroid imaging demonstrated improved tumor infiltration and lysis (Figure 7g,h; Figure S8d–f). We next asked whether the observed mitochondrial effects could be explained by off–target modulation of NF‐κB, given the reported crosstalk between MAPK and NF‐κB pathways [39]. Inhibition of NF‑κB with BMS‑345541, however, did not abolish the MAPKi‑associated increase in mitochondrial abundance, indicating that this mitochondrial phenotype is primarily attributable to MAPK inhibition by sesamolin (Figure S9a–d).

We next evaluated MAPKi‐pretreated iNK cells in vivo using an A549‐Luc xenograft model. Mice received three intravenous injections of WT or MAPKi‐pretreated iNK cells on days 7, 13, and 21 without exogenous IL‐2 (Figure S10a). Both iNK groups suppressed tumor growth, with stronger tumor control in the MAPKi group, without evident body‐weight loss (Figure S10b–e). At 7 and 14 d after the final infusion, MAPKi‐pretreated iNK cells were detected at higher frequencies than WT iNK cells in peripheral blood and tumor tissues, indicating improved persistence and tumor infiltration (Figure S10f,g). Together, these results show that MAPK attenuation enhances hypoxia tolerance, mitochondrial fitness, antitumor activity, and in vivo persistence across both NK92 and iPSC‐derived iNK platforms.

## 3 Discussion

Solid tumor immunotherapy faces a fundamental challenge: although immune cells can be genetically engineered to recognize malignant cells, their therapeutic efficacy critically depends on their ability to function within the spatially heterogeneous, metabolically hostile, and immunosuppressive tumor microenvironment following adoptive transfer [7, 22]. Thus, durable antitumor activity requires not only tumor recognition but also environmental fitness. Our study reframes hypoxia—a pervasive suppressive feature of solid tumors—not as an immutable constraint, but as a tunable physiological cue that can be harnessed for immune cell conditioning [40, 41]. By progressively reducing oxygen tension from 5% to 1% O_2_, hypoxic preconditioning (HPC) enabled NK cells to acquire resistance to subsequent acute hypoxic stress, preserving mitochondrial respiration, ATP production, cytotoxic effector function, and tumor‐spheroid infiltration. These findings indicate that hypoxia‐induced NK cell dysfunction is not necessarily an unavoidable consequence of low oxygen exposure, but depends on the kinetics of hypoxic stress and can be attenuated by gradual hypoxic preconditioning. This distinction further raises a key mechanistic question: what determines whether hypoxic stress drives NK cell injury or is redirected into adaptive fitness?

To gain mechanistic insight into HPC‐mediated protection, we interpreted the HPC versus AH pathway‐enrichment profile. GSEA identified ErbB and MAPK as the two highest‐ranked enriched pathways. ErbB signaling is known to activate downstream MAPK and PI3K‐Akt cascades [42], whereas several other enriched pathways, including cAMP/Rap1, GnRH, NF‐κB, C‐type lectin receptor, and HIF‐1α signaling, have been reported to interact with MAPK‐related signaling networks [43, 44, 45, 46]. Together, this enrichment pattern suggests that MAPK may represent a central stress‐responsive signaling axis in hypoxia‐exposed NK cells. Furthermore, by clinical single‐cell transcriptomic analysis, tumor‐infiltrating NK cells from NSCLC patients exhibited enriched MAPK‐related transcriptional programs compared with NK cells from adjacent normal tissues. We therefore focused subsequent mechanistic analysis on MAPK signaling, particularly the stress‐responsive p38 and JNK branches.

Biochemical validation extended these observations beyond transcript‐level associations: decreasing oxygen concentration progressively increased phosphorylation of p38 MAPK and JNK, whereas HPC attenuated this stress‐responsive activation. Pharmacological activation of stress‐MAPK signaling with anisomycin under normoxia induced mitochondrial ROS, partially recapitulating the mitochondrial stress phenotype observed under acute hypoxia, while MAPK reactivation partially reversed the protective effects of HPC. This model is conceptually reminiscent of hypoxic preconditioning in the nervous system, where brief non‐lethal hypoxia induces ischemic tolerance through HIF‐dependent transcriptional adaptation, controlled mitochondrial ROS signaling, antioxidant defense, and mitochondrial quality control [47, 48]. However, unlike neurons, NK cells must preserve cytotoxic effector fitness rather than merely acquire survival tolerance. This interpretation is further supported by recent antitumor T‐cell studies showing that p38 inhibition enhances tumor‐reactive and gene‐engineered T‐cell function, suggesting that stress‐MAPK signaling can act as a druggable checkpoint of cytotoxic lymphocyte fitness [49]. Together, these findings position p38/JNK activation as a context‐dependent threshold signal that permits mitochondrial adaptation when restrained, but drives mitochondrial injury and NK cell dysfunction when sustained.

Mechanistically, the protective effects of HPC and MAPK attenuation were not limited to hypoxia resistance alone, but involved a broader remodeling of NK cell fitness. MAPK attenuation appears to reset a broader stress‒mitochondrial adaptation program in which *PPARGC1A* contributes functionally to mitochondrial remodeling. Transcriptomic profiling revealed upregulation of *PPARGC1A* following MAPK attenuation, consistent with activation of a PGC‐1α‒associated mitochondrial remodeling program, while PGC‐1α depletion diminished the associated improvements in mitochondrial respiration and redox control. These findings suggest that PGC‐1α represents an important component of a wider adaptive state that also includes preservation of respiratory reserve, limitation of mitochondrial stress amplification, and improved energetic readiness before hypoxic exposure. In parallel, HPC attenuated transcriptional programs linked to TME‐mediated immunosuppression, including adenosine‒A2A receptor signaling, immune checkpoint pathways, and TGF‐β/SMAD signaling.

In addition to improving metabolic and cytotoxic fitness, HPC and MAPK modulation also promoted NK cell trafficking and persistence in tumor models. Effective NK cell therapy for solid tumors requires not only robust cytolytic capacity but also efficient tumor homing, intratumoral retention, and sustained functional competence within hostile tissue niches [27]. AH significantly downregulated CXCR6 and CD69 expression in NK92 cells—whereas pretreatment with HPC or MAPKi restored this trafficking‐ and retention‐associated program and was accompanied by increased NK cell accumulation in both lung cancer spheroids and tumor tissues. Collectively, these results indicate that metabolic adaptation supports antitumor activity through multiple complementary mechanisms, including improved mitochondrial fitness, attenuation of immunosuppressive transcriptional programs, preservation of cytotoxic function under hypoxic stress, and enhanced spatial persistence within the tumor parenchyma.

A key translational implication of this work is that the adaptive state revealed by HPC can be pharmacologically mimicked. Pretreatment with a MAPK inhibitor under normoxia recapitulated core protective features of HPC—including enhanced mitochondrial biogenesis, improved metabolic flexibility, and restoration of NK92 antitumor function under hypoxic challenge. Consistently, systemic MAPK inhibitor administration promoted mitochondrial remodeling in endogenous peripheral‐blood NK cells in immunocompetent BALB/c mice, further supporting the in vivo relevance of MAPK modulation for NK cell metabolic fitness. Importantly, sesamolin is a sesame‐derived lignan closely related to sesamin, a clinically investigated sesame lignan. Sesamin has been investigated clinically in patients with type 2 diabetes and inflammatory conditions, demonstrating a favorable safety profile [50, 51]. Nevertheless, its potential application in immune‐cell modulation and cancer immunotherapy has not been systematically explored. Our findings provide a rationale for further preclinical and translational studies to determine whether sesamolin‐mediated MAPK modulation can improve NK cell metabolic fitness and antitumor function in hypoxic solid tumors.

Importantly, the protective effect of MAPK modulation was not restricted to NK92 cells. Although NK92 cells provide a tractable and reproducible model for dissecting hypoxia‐induced mitochondrial injury and stress‐MAPK signaling, their lymphoma‐derived origin and cell‐line‐specific biology limit direct extrapolation to therapeutic NK products [52, 53]. We therefore validated the key findings in human iPSC‐derived induced NK cells. Consistent with the NK92 data, MAPKi treatment also improved mitochondrial function and enhanced cytotoxic activity in iNK cells under hypoxic stress, indicating that the MAPK‒mitochondrial checkpoint is not merely a feature of NK92 cells but is operative across distinct NK cell platforms. This is particularly relevant because iNK cells are being developed as scalable, off‐the‐shelf products for cancer immunotherapy [54], and the enhanced tumor control and persistence observed after adoptive transfer further support the potential translational relevance of this metabolic‐priming strategy. In this framework, stress‐adaptation engineering may represent an additional layer of NK cell product optimization, aimed at improving persistence and functional durability after infusion.

Collectively, our study reframes tumor hypoxia from a purely suppressive barrier into a programmable conditioning cue. HPC reveals that NK cells can be metabolically conditioned to tolerate severe hypoxic stress, whereas MAPK attenuation provides a faster and more pharmacologically tractable approach to reproduce key features of this adaptive state. By defining a modifiable p38/JNK‐centered MAPK‒mitochondrial checkpoint that links hypoxic stress to NK cell dysfunction, our work provides a conceptual and practical framework for improving NK cell environmental fitness in solid tumors. This strategy may help shift NK cell engineering beyond tumor recognition alone toward integrated optimization of recognition capacity, metabolic resilience, and tissue persistence. One limitation should be noted. Although MAPKi administration in BALB/c mice increased NKp46 expression on endogenous NK cells, this in vivo experiment does not distinguish whether MAPK attenuation acts directly on NK cells or indirectly through other immune populations, such as macrophages and dendritic cells. Future studies using NK cell‐specific perturbation or adoptive‐transfer models will be needed to define the cell‐intrinsic contribution of MAPK attenuation in vivo. Furthermore, the associations of HIF‐1α expression and MAPK activation with NK cell functional status and lung cancer patient survival remain to be validated at the protein level in human lung cancer tissues. Future studies integrating multiplex immunofluorescence staining with well‐annotated clinical cohorts will be important to clarify these associations and their prognostic significance.

## 4 Methods

### 4.1 Cell Lines and Culture Conditions

A549, PC‐9, H1299, and 293T cell lines were obtained from the American Type Culture Collection (ATCC; A549 RRID: CVCL_0023; PC‐9 RRID: CVCL_B260; H1299 RRID: CVCL_0060; 293T RRID: CVCL_0063). NK92 cells were purchased from Procell Life Science & Technology (RRID: CVCL_2142), and the human induced pluripotent stem cell line ML18 was obtained from UniXell Biotechnology. A549, PC‐9 and H1299 cells were cultured in RPMI‐1640 medium (Thermo Fisher Scientific, 11875119) supplemented with 10% fetal bovine serum (ExCell, FSP500) and penicillin–streptomycin (Thermo Fisher Scientific, 15140122). 293T cells were maintained in DMEM (Thermo Fisher Scientific, 11995‐065) supplemented with 10% fetal bovine serum and penicillin–streptomycin. NK92 cells were cultured in NK92 Complete Medium (Procell, CM‐0530). ML18 cells were maintained in mTeSR Plus medium (STEMCELL Technologies, 100–0276). All cells were maintained at 37°C in a humidified incubator with 5% CO_2_. All cell lines were routinely tested for mycoplasma contamination.

### 4.2 Animal Studies

M‐NSG mice used for the NK92 xenograft experiments were purchased from Shanghai Model Organisms Center, Inc. (SMOC), whereas M‐NSG mice used for the iNK xenograft experiments were obtained from GemPharmatech Co., Ltd. BALB/c mice used for the systemic MAPKi experiments were also obtained from GemPharmatech Co., Ltd. Animals were housed at Guangzhou National Laboratory under specific pathogen‐free (SPF) conditions, with no more than six mice per cage, a 12 h light/12 h dark cycle, and free access to standard laboratory chow and water. Experimental mice were 8–18 weeks of age and weighed 18–25 g at the time of use. Both male and female mice were used, and all mice within each individual experiment were of the same sex.

Following confirmation of tumor establishment by bioluminescence imaging, mice were randomly assigned to treatment groups with comparable baseline tumor burdens. For the NK92 xenograft experiment shown in Figure 6, mice were assigned to the PBS group (*n* = 3) or to the WT NK92, MAPKi‐pretreated NK92, and HPC‐pretreated NK92 groups (*n* = 7 per group). For the systemic MAPKi experiment in BALB/c mice shown in Figure 4, 12 mice were randomly assigned to vehicle and MAPKi treatment groups (*n* = 6 per group). For the iNK xenograft experiment, mice were randomly assigned to the PBS group (*n* = 3) or to the WT iNK and MAPKi‐pretreated iNK groups (*n* = 6 per group). The exact number of animals analyzed for each endpoint is indicated in the corresponding figure legends. Differences in sample size among panels reflect endpoint‐specific sample availability and/or the use of subsets of animals for downstream analyses.

Tumor growth and body weight were monitored throughout the experiments. A tumor volume exceeding 2000 mm^3^ was predefined as a humane endpoint. Mice were also euthanized if they exhibited other predefined signs of severe distress or clinical deterioration, including excessive body‐weight loss, loss of ambulation or mobility, inability to access food or water, or marked deterioration of general condition. All animals meeting any humane endpoint were euthanized in accordance with the approved institutional protocol. All animal procedures were approved by the Institutional Animal Care and Use Committee of Guangzhou National Laboratory (Approval No. GZLAB‐AUCP‐2026‐03‐A13).

### 4.3 Differentiation of iPSC‐Derived NK Cells and Phenotype Analysis

The human induced pluripotent stem cell line ML18 was maintained in mTeSR Plus medium (STEMCELL Technologies, 100–0276) on plates coated with recombinant human vitronectin (Gibco, A14700). Differentiation of iPSCs into NK cells was performed using the STEMdiff NK Cell Kit (STEMCELL Technologies, 100–0170) according to the manufacturer's instructions, which includes embryoid body (EB) formation, isolation of CD34^+^ hematopoietic progenitor cells, and subsequent NK cell differentiation. During the differentiation process, cellular phenotype was evaluated by flow cytometry. Cells were stained with PE‐conjugated anti‐human CD34 (BioLegend, 343506), BV421‐conjugated anti‐human CD45 (BD Biosciences, 563879), PE‐conjugated anti‐human CD56 (BioLegend, 304606), APC‐conjugated anti‐human CD16 (BioLegend, 302012), and FITC‐conjugated anti‐human CD3 (BD Biosciences, 555339). Data were acquired and analyzed using an Agilent NovoCyte flow cytometer.

### 4.4 Hypoxic Preconditioning and Acute Hypoxic Challenge

Hypoxic preconditioning (HPC) was performed using a graded oxygen‐reduction protocol. NK92 cells were cultured sequentially at 5%, 3%, and 1% O_2_, with each oxygen condition maintained for 48 h. After completion of the graded hypoxic preconditioning regimen, cells were returned to normoxic culture conditions for 48 h before subsequent experimental use. For acute hypoxic challenge, NK92 cells were exposed directly to 1% O_2_ for 36 h. Normoxic control, AH, and HPC groups were cultured in parallel and processed at matched experimental time points to ensure synchronized comparison. Hypoxic conditions were generated using the Cell+100 intelligent cell preparation system, with oxygen tension set to 5%, 3%, or 1% O_2_ as required.

### 4.5 IncuCyte Live‐Cell Cytotoxicity Assay

A549, H1299, and PC‐9 tumor cells were engineered to stably express tdTomato and luciferase (A549‐tdTomato‐Luciferase, H1299‐tdTomato‐Luciferase, and PC‐9‐tdTomato‐Luciferase). For 2D cytotoxicity assays, tumor cells were seeded in 96‐well flat‐bottom plates at 8,000 cells per well and cultured overnight before the addition of NK cells at an effector‐to‐target (E:T) ratio of 4:1. Cytotoxicity was monitored using the IncuCyte S3 live‐cell imaging system, with images acquired every 30 min or 1 h for 24 h, and target cell viability was quantified by measuring the red fluorescent cell area.

For 3D spheroid assays, tdTomato‐expressing tumor cells were seeded at 4,000 cells per well in 200 µL complete medium in ultra‐low attachment round‐bottom 96‐well plates (Corning, 7007). Plates were centrifuged at 400 × g for 5 min to facilitate spheroid formation and incubated at 37°C with 5% CO_2_ for 72 h. NK cells were subsequently added at an E:T ratio of 8:1. Spheroid growth and NK cell‐mediated cytotoxicity were monitored using the IncuCyte S3 system with a 10× objective, with images acquired every 2 h for 48 h. All experiments were performed with at least three independent replicates.

### 4.6 Oxidative Phosphorylation and Glycolytic Rate Assays

Metabolic activity of NK cells was assessed using Seahorse extracellular flux analysis. Enriched NK cells were resuspended in Seahorse XF Assay Medium (Agilent Technologies). Seahorse XF96 plates were precoated with 0.1 mg mL^−1^ poly‐L‐lysine (Feimobio Life Sciences, FB29984) to facilitate cell attachment, and 100 000 NK cells were seeded per well to form a uniform monolayer. The oxygen consumption rate (OCR) and extracellular acidification rate (ECAR) were measured in real time using an XFe96 Extracellular Flux Analyzer (Agilent Technologies). Mitochondrial respiration was assessed using the Seahorse XF Mito Stress Test Kit, with sequential injections of oligomycin (1.5 µm), FCCP (1 µm), and rotenone/antimycin A (0.5 µm). Glycolytic activity was measured using the Seahorse XF Glycolytic Rate Assay Kit, with sequential addition of rotenone/antimycin A followed by 2‐deoxy‐D‐glucose (50 mm). OCR values were derived from the Mito Stress Test and ECAR values from the Glycolytic Rate Assay.

### 4.7 Mitochondrial Function Assays

Mitochondrial status and metabolic activity of NK cells were evaluated using MitoTracker staining, mitochondrial ROS measurement, and ATP quantification. For mitochondrial imaging, cells were stained with 200 nM MitoTracker Red CMXRos (Yeasen, 40741ES50) and Hoechst 33342 (Invitrogen, R37165) for 30 min at 37°C, followed by imaging using a ZEISS LSM 900 confocal microscope equipped with Airyscan and a 63× objective. Mitochondrial reactive oxygen species (ROS) levels were measured using MitoSOX Red Mitochondrial Superoxide Indicator (MedChemExpress, HY‐D1055). Briefly, 5 × 10^5^ NK cells per condition were stained with 5 µm MitoSOX for 30 min and immediately analyzed using an Agilent NovoCyte flow cytometer. Cellular ATP levels were determined using an Enhanced ATP Assay Kit (Beyotime, S0027) according to the manufacturer's instructions. Luminescence signals were measured using a BioTek Synergy Neo2 multimode microplate reader.

### 4.8 In Vivo Xenograft Studies

A549‐Luc cells were cultured at 37°C with 5% CO_2_, harvested, and resuspended in 100 µL DPBS (Thermo Fisher Scientific, 14190250). A549‐Luc cells (5 × 10^6^ cells per mouse) were subcutaneously injected to establish xenograft tumors. Seven days after tumor inoculation, tumor burden was assessed by bioluminescence imaging (BLI), and mice were assigned to treatment groups with comparable BLI signal intensities before receiving intravenous injections of NK92 or iPSC‐derived NK (iNK) cells. For mice receiving NK92 cells, IL‐2 (NOVOPROTEIN, GMP‐C013) was administered every 2‒3 d for a total of 21 d to support NK92 cells. No exogenous IL‐2 was administered to mice receiving iNK cells. Tumor growth was monitored every 2 d by digital caliper measurement, and body weight was recorded at the same intervals. Tumor volume was calculated as *V* = *L* × *W* ^2^ × 0.5, where *L* represents the longest tumor diameter and *W* represents the perpendicular diameter. Mice were euthanized when tumors reached the maximum allowable size or when predefined humane endpoints, including loss of ambulation, were reached.

### 4.9 In Vivo MAPK Modulation in BALB/c Mice

To evaluate the effect of systemic MAPK modulation on endogenous NK cells, twelve male BALB/c mice aged 6–8 weeks were randomly assigned to vehicle control and MAPKi treatment groups (*n* = 6 per group). Mice in the MAPKi group received intraperitoneal injections of sesamolin (6 mg kg^−1^), whereas control mice received an equal volume of vehicle. Injections were administered three times at 48‐h intervals, and peripheral blood was collected 48 h after the final injection. BALB/c mice were obtained from GemPharmatech, and all procedures were performed in accordance with protocols approved by the Institutional Animal Care and Use Committee of Guangzhou National Laboratory.

For in vivo analysis of NK cell persistence and tumor infiltration, peripheral blood was collected from anesthetized NSG mice via retro‐orbital bleeding, followed by red blood cell lysis using lysing buffer (BD Biosciences, 555899). Tumor tissues were excised and enzymatically dissociated in PBS containing Collagenase IV (Dogesce, BG349), hyaluronidase (Dogesce, BG355), and DNase I (Thermo Fisher, EN0521) to generate single‐cell suspensions. Blood‐ and tumor‐derived cells were subsequently stained with PE‐conjugated anti‐human CD56 (BioLegend, 304606) together with APC‐conjugated anti‐human CD45 (Selleck, H0093) or PE/Cy7‐conjugated anti‐human CD45 (BioLegend, 368532). For intracellular phospho‐MAPK analysis, cells were fixed and permeabilized and subsequently stained with Alexa Fluor 488‐conjugated anti‐phospho‐p38 MAPK (Thr180/Tyr182) (BioLegend, 690255). Flow cytometry data were acquired using an Agilent NovoCyte flow cytometer.

For ultrastructural analysis of endogenous NK cells, peripheral blood cells following red blood cell lysis were stained with PE‐conjugated anti‐mouse CD49b and DAPI. Live CD49b^+^ cells were isolated by fluorescence‐activated cell sorting using a Sony MA900 Cell Sorter (LE‐MA900FP) and collected for subsequent transmission electron microscopy (TEM) analysis.

### 4.10 RNA‐Seq Sample Collection and Data Analysis

NK92 cells from the indicated treatment groups were collected, and total RNA was extracted. RNA purity and concentration were assessed using a NanoDrop 2000 spectrophotometer, and RNA integrity was evaluated using the LabChip GX Touch system. Qualified RNA samples were used for library construction. mRNA was enriched using oligo (dT) magnetic beads, fragmented, and reverse‐transcribed into cDNA. After end repair, A‐tailing, adapter ligation, size selection, and PCR amplification, sequencing libraries were generated. Library quality was assessed using Qubit 3.0, an Agilent 2100 Bioanalyzer, and qPCR, followed by Illumina sequencing to generate 150‐bp paired‐end reads.

Raw reads were filtered to remove adapter‐containing, low‐quality, and undetermined‐base reads. Clean reads were aligned to the reference genome using HISAT2 v2.0.5. Gene expression levels were quantified using read counts and FPKM values. Differential expression analysis was performed using DESeq2 v1.46.0 in R v4.4.2, with Benjamini–Hochberg‐adjusted *p* ≤ 0.05 considered statistically significant. GO and KEGG enrichment analyses were performed using clusterProfiler v4.14.6. GSEA was performed using clusterProfiler v4.14.6 and fgsea v1.32.4 based on ranked gene lists generated from differential expression statistics. Normalized expression matrices were further processed using limma v3.62.2 for downstream visualization where indicated. Pathways related to hypoxia response, MAPK signaling, mitochondrial metabolism, glycolysis, and NK cell effector function were further extracted for focused analysis.

### 4.11 Statistical Analysis

Data are presented as mean ± SD. Statistical analyses were performed using IBM SPSS Statistics, except for longitudinal 3D tumor spheroid cytotoxicity assays, which were analyzed using GraphPad Prism. At least three independent experiments were performed for statistical analysis. The number of samples in different experiments is shown in the figure legends. Comparisons between two independent groups were performed using an unpaired two‐tailed Student's *t*‐test, whereas comparisons among three or more groups were analyzed using one‐way ANOVA followed by Tukey's multiple‐comparison tests. Longitudinal 3D tumor spheroid cytotoxicity data were analyzed using two‐way ANOVA followed by Tukey's multiple‐comparison tests. Survival curves were analyzed using the Kaplan–Meier method and compared using the log‐rank test. All tests were two‐sided, and *p* < 0.05 was considered statistically significant.

## Author Contributions

H.L. conceived the project. H.L., J.S., W.Z., and T.X. supervised the research. D.Z., X.Z., N.W., and Q.L. performed the experiments. D.Z., X.Z., Q.L., and L.S. conducted the animal experiments. Y.Y., S.H., and N.W. differentiated iNK cells. D.Z., X.Z., and C.M. analyzed the data and wrote the manuscript. The authors declare no other competing financial interests.

## Funding

This work was supported by a Major Project of Guangzhou National Laboratory (Grant No. GZNL2024A03012 to H.L.), Guangzhou National Laboratory and State Key Laboratory of Respiratory Disease (GZNL2025B01010 to H.L.).

## Ethics Statement

All animal procedures were approved by the Institutional Animal Care and Use Committee of Guangzhou National Laboratory (Approval No. GZLAB‐AUCP‐2026‐03‐A13). All mice were housed and maintained at Guangzhou National Laboratory under specific pathogen‐free conditions, and all animal experiments were conducted in accordance with the approved institutional guidelines.

## Conflicts of Interest

The authors declare no conflicts of interest.

## Supporting information




**Supporting File**: advs78759‐sup‐0001‐SuppMat.docx.

## Data Availability

All data supporting the findings of this study are included in the article and its Supporting Information. Publicly available single‐cell transcriptomic data analyzed in this study are available from the Gene Expression Omnibus under accession numbers GSE198099, GSE72094, and GSE131907. The RNA‐sequencing data generated in this study have been deposited in the NCBI Sequence Read Archive under BioProject accession PRJNA1520324. Additional data supporting the findings of this study are available from the corresponding author upon reasonable request.
