# Supplementary material for: Hypoxic Preconditioning Restores NK Cell Metabolic Fitness by Attenuating MAPK Signaling to Potentiate Antitumor Immunity in Lung Cancer
Source: Adv Sci (Weinh). 2026 Nov 9:e78759. Online ahead of print. doi: 10.1002/advs.78759 (PMC13748898; doi:10.1002/advs.78759)
Supplement: Supplementary file 1 [file ADVS-9999-e78759-s001.docx]

Supplementary Materials for

Hypoxic preconditioning restores NK cell metabolic fitness by attenuating MAPK signaling to potentiate antitumor immunity in lung cancer

*Dian Zhuang^#^, Xiaoyue Zhu^#^, Na Wang, Qianqian Liu, Lihua Chen, Yanzhuo Ye, Lingrui Shang, Shasha Huang, Cong Ma, Wei Kevin Zhang*, Tao Xu*, Jiaqi Sun*, Huisheng Liu**

Correspondence to:

liu_huisheng@gzlab.ac.cn; [sun_jiaqi@gzlab.ac.cn;](mailto:sun_jiaqi@gzlab.ac.cn;) [xu_tao@gzlab.ac.cn;](mailto:xu_tao@gzlab.ac.cn;) zhang_wei@gzlab.ac.cn

**This PDF file includes:**

Methods

Figure S1 to S10

Table S1 and S2

**Methods**

**RNA extraction and quantitative real-time PCR (qRT-PCR)**

Total RNA was extracted from cells using the Cell Total RNA Rapid Extraction Kit (GOONIEBIO, 400-100) according to the manufacturer’s instructions. Briefly, cells were lysed and total RNA was purified using the supplied silica-based spin columns, followed by washing and elution with RNase-free elution buffer. The extracted RNA was used for subsequent reverse transcription. First-strand cDNA was synthesized using the Fast First-Strand cDNA Synthesis Mix for RT (with dsDNase) (GOONIEBIO, 500-101) according to the manufacturer’s instructions. Genomic DNA was removed by dsDNase treatment prior to reverse transcription, and 50 ng‒1 μg total RNA was used in a 20 μL reaction. The resulting cDNA was used as the template for quantitative real-time PCR.

Quantitative real-time PCR was performed using Fast Taq Template Visualization SYBR Green qPCR Mix (GOONIEBIO, 500-100) according to the manufacturer’s instructions. The 2× SYBR Green master mix was used at a final concentration of 1× together with gene-specific primers and cDNA template. Relative gene expression was calculated using the 2^−ΔΔCt method, with β-Actin as the internal reference gene. Primer sequences are provided in Supplementary Table 2.

**Western blot**

NK92 cells were lysed in RIPA (Beyotime, P0013B) buffer supplemented with a protease inhibitor cocktail (MedChemExpress, HY-K0010). Protein concentrations were determined using the BCA Protein Assay Kit (Beyotime, P0012). Equal amounts of protein (50 μg) were separated by SDS-PAGE using precast gels (GenScript, M00610) and transferred onto PVDF membranes (Cytiva, 10600023). Membranes were blocked with blocking buffer (Beyotime, P0023B) for 1 h at room temperature and incubated overnight at 4 °C with primary antibodies against β-Actin (Proteintech, 66009-1-Ig), HIF-1α (Selleck, F0101), p38 MAPK (Cell Signaling Technology, 8690T), SAPK/JNK (Cell Signaling Technology, 9252T), phospho-p38 (Thr180/Tyr182) (Cell Signaling Technology, 4511T), phospho-SAPK/JNK (Thr183/Tyr185) (Cell Signaling Technology, 4668T), phospho-NF-kappaB p65 (Ser536) (Cell Signaling Technology, 3033T), NF-kappaB p65 (Cell Signaling Technology, 8242T), phospho-p44/42 MAPK (Erk1/2) (Thr202/Tyr204) (Cell Signaling Technology, 4370T), and p44/42 MAPK (Erk1/2) (Cell Signaling Technology, 4695). After washing with TBST (Sangon Biotech, C520009-0500), membranes were incubated with HRP-conjugated anti-mouse or anti-rabbit IgG antibodies (Proteintech, SA00001-1 or SA00001-2) for 1.5 h at room temperature. Signals were detected using the BeyoECL Moon Kit (Beyotime, P0018FM) and imaged with a Tanon 5200 chemiluminescence imaging system. Antibody information and working dilutions are provided in Supplementary Table 1.

**siRNA-mediated gene knockdown**

For transient gene knockdown, NK92 cells were transfected with siRNAs targeting *PPARGC1A*, *MAPK8*, or *MAPK14*, or with a non-targeting negative-control siRNA (siNC) (Tsingke Biotechnology, Beijing, China), at a final concentration of 100 nM using Lipofectamine 3000 (Invitrogen, L3000015) in Opti-MEM I Reduced Serum Medium (Gibco, 31985070), according to the manufacturer’s instructions.

For PGC-1α loss-of-function experiments, cells were assigned to four groups: siNC, siNC + sesamolin (MAPKi), and siPPARGC1A+sesamolin (siPPARGC1A+i). siRNA transfection and sesamolin treatment (5 μM) were initiated simultaneously under normoxic conditions. After 48 h, cells were collected for qPCR to confirm *PPARGC1A* knockdown and for subsequent extracellular flux analyses.

For genetic attenuation of stress-MAPK signaling, cells were transfected with either siNC or a combination of siRNAs targeting *MAPK8* (JNK1) and *MAPK14* (p38α). Twenty-four hours after transfection, cells were exposed to 1% O_2_ for an additional 24 h. At 48 h after transfection, cells were collected for qPCR to confirm *MAPK8* and *MAPK14* knockdown and for ROS and extracellular flux analyses. The siRNA sequences are provided in Supplementary Table 2.

**Pharmacological modulation of MAPK, NF-κB, and HIF-1α signaling**

Sesamolin was used as a MAPK-modulating compound. Sesamolin was purchased from MedChemExpress (MedChemExpress, HY-N0809) and prepared as a stock solution in DMSO. For MAPK attenuation experiments, NK92 cells were pretreated with sesamolin at a final concentration of 5 μM under normoxic conditions for 48 h. PBS-treated cells were used as vehicle controls. For combined MAPK and NF-κB modulation, BMS-345541 (MedChemExpress, HY-10519) was added at a final concentration of 0.5 μM together with 5 μM sesamolin during the pretreatment period. After pretreatment, cells were washed with PBS to remove residual compounds and were then subjected to an acute hypoxic challenge at 1% O_2_ for 36 h where indicated. For MAPK activation experiments, NK92 cells were treated with anisomycin (Selleck, S7409) at 2 μM for 30 min under normoxic conditions. For HIF-1α inhibition experiments, NK92 cells were treated with PX-478 (Selleck, S7612) at 20 μM for 48 h under 1% O_2_. After drug treatment, cells were collected for downstream analyses, including western blotting, mitochondrial ROS detection, metabolic assays, flow cytometry, and cytotoxicity assays.

**Cell proliferation and cell viability assessment**

For EdU (5-ethynyl-2’-deoxyuridine) assay, logarithmic growth phase cells were seeded in 12-well plates with corresponding concentration of EdU reagent for 2 h. Cells were washed with PBS for 5 min twice, before incubating with 4% Paraformaldehyde for 30 min. After washing with PBS for 5 min twice, samples were permeabilized with 0.3% TritonX-100 in PBS, and stained with reaction solution (Beyotime, C0075S). Images were acquired by flow cytometry on an Agilent NovoCyte^TM^ flow cytometer.

The viability of cells was determined using the trypan blue dye (Invitrogen, 15250061) exclusion method. Ten microliters (10 µL) of the cell suspension was mixed with an equal volume of 0.4% (w/v) trypan blue solution, and the mixture was carefully loaded into a disposable counting slide and analyzed using an automated cell counter (HUAPO) according to the manufacturer's instructions. The instrument automatically calculated the total cell concentration and viability percentage.

**Lentivirus production**

Lentiviral particles encoding tdTomato–Luciferase were generated by transient co-transfection of HEK293T cells with the transfer vector and the packaging plasmids psPAX2 and pMD2.G. Viral supernatants were collected at 48 h and 60 h post-transfection and filtered through a 0.45 μm filter to remove cellular debris. For concentration, 5× TransLv™ Lentivirus Precipitation Solution (TransGen, FV101-01) was added to the viral supernatant to a final concentration of 1×, mixed gently, and incubated at 4°C for 45 min. The mixture was then centrifuged at 7,000 × g for 45 min at 4°C. The viral pellet was resuspended in PBS and stored at −80°C until use.

**Flow cytometry**

To assess NK cell activation and functional marker expression, NK92 cells were co-cultured with A549 tumor cells at an effector-to-target (E:T) ratio of 4:1. After incubation, cells were stained with the following antibodies for a 20 min staining period at 4°C and protected from light: PE-conjugated anti-human CD107a (BioLegend, 328608), APC-conjugated anti-human FasL (BioLegend, 306422), PE-conjugated anti-human CD69 (BioLegend, 310906), PE-conjugated anti-human NKG2D (BioLegend, 320806), APC-conjugated anti-human NKG2A (BioLegend, 375108), BV785-conjugated anti-human NKG2A (BioLegend, 375137), APC-conjugated anti-human NKp46 (BioLegend, 331918), BV421-conjugated anti-human TRAIL (BioLegend, 308221), APC-conjugated anti-human TRAIL (BioLegend, 308210), PE-conjugated anti-human CXCR6 (BioLegend, 356003)，APC-conjugated anti-human CD25 (BioLegend, 302609), PE-conjugated anti-human CD122 (BioLegend, 339005), PE-conjugated anti-human CD132 (BioLegend, 338605), BV421-conjugated Mouse IgG1, κ isotype control (BioLegend, 400157), PE-conjugated Mouse IgG1, κ isotype control (BioLegend, 400111), PerCP/Cy5.5-conjugated Mouse IgG1, κ isotype control (BioLegend, 400149), and APC-conjugated Mouse IgG1, κ isotype control (BioLegend, 400120). Cells were subsequently stained with DAPI (BD Biosciences, 564907) for 10 min to exclude dead cells. For intracellular staining, cells were fixed and permeabilized prior to staining with PerCP/Cy5.5-conjugated anti-human T-bet (BioLegend, 365907) or PE-conjugated anti-human T-bet (BioLegend, 365903).

For intracellular Granzyme B staining, NK cells were co-cultured with A549 target cells at an effector-to-target (E:T) ratio of 4:1 for 4 h. Brefeldin A (5 μg/mL; Yeasen Biotechnology, 50502ES03) and Monensin Solution (1000×; Yeasen Biotechnology, 50501ES03) were added during the 4 h co-culture period to inhibit protein secretion and permit intracellular accumulation of Granzyme B. After co-culture, cells were harvested and fixed/permeabilized using the BD Pharmingen Transcription Factor Buffer Set (BD Biosciences, 562574) according to the manufacturer’s instructions. Following fixation and permeabilization for 45 min, cells were stained with APC-conjugated anti-human/mouse Granzyme B recombinant antibody (BioLegend, 372203) and analyzed by flow cytometry. Antibody information and working dilutions are provided in Supplementary Table 1.

**Cytotoxicity assay**

NK cell cytotoxic activity was evaluated by measuring the secretion of effector molecules and tumor cell viability. NK cells were co-cultured with tumor cells at an effector-to-target (E:T) ratio of 4:1 for 4 h. Culture supernatants were then collected and analyzed using Human Granzyme B ELISA Kit (Proteintech, KE00121), Human Perforin-1 ELISA Kit (MLBio, ml105197), and Human IFN-γ ELISA Kit (Elabscience, E-HSEL-H0007) according to the manufacturers’ instructions.

**Transmission electron microscopy**

NK92 cells from the indicated treatment groups were collected by centrifugation and washed twice with cold PBS. Cell pellets were fixed in 2.5% glutaraldehyde in 0.1 M phosphate buffer at 4 °C overnight. After fixation, samples were washed with 0.1 M phosphate buffer and post-fixed with 1% osmium tetroxide for 1–2 h at room temperature. The samples were then dehydrated through a graded ethanol series, infiltrated with epoxy resin, and embedded for polymerization. Ultrathin sections of approximately 70 nm were prepared using an ultramicrotome, mounted on copper grids, and stained with uranyl acetate and lead citrate. Mitochondrial ultrastructure was examined using a Talos L120C transmission electron microscope.

**Figure S1.**

**Figure S1. Acute hypoxia suppresses NK92 cell cytotoxicity against lung cancer cells.**

**(a)** Kaplan–Meier overall survival curves for patients with high and low HIF-1α expression in the GSE72094 cohort, stratified according to the median expression level. The log-rank p value is shown. **(b)** Forest plot of the multivariable Cox proportional hazards model evaluating HIF-1a expression as a continuous standardized variable, adjusted for age, sex, and tumor stage. Hazard ratios and 95% confidence intervals are shown.

**(c)** Cytotoxic activity of WT- and AH-treated NK92 cells against lung cancer cell lines PC-9-tdTomato-Luciferase and H1299-tdTomato-Luciferase, evaluated using the IncuCyte real-time imaging system (E:T = 4:1; n = 3).

**(d)** Cytotoxic activity of NK92 cells against PC-9-tdTomato-Luciferase and H1299-tdTomato-Luciferase 3D tumor spheroids, evaluated using the IncuCyte real-time imaging system. Left panel: Representative red fluorescence images at indicated time points; scale bar = 200 μm. Right panel: Corresponding quantitative analysis (E:T = 8:1; n = 3).

**(e)** Cytotoxicity of WT- and AH-treated NK92 cells against A549-tdTomato-Luciferase, PC-9-tdTomato-Luciferase, and H1299-tdTomato-Luciferase was assessed by luciferase luminescence imaging. Blue and red regions represent the areas of adherent viable cells (E:T = 4:1; time = 4 h; n = 3).

Data are presented as mean ± SD where applicable. Statistical analyses were performed using IBM SPSS Statistics, except for the 3D tumor spheroid cytotoxicity assay in panel (d), which was analyzed using GraphPad Prism. Overall survival in panel (a) was analyzed using the Kaplan‒Meier method and compared using the log-rank test. Multivariable survival analysis in panel (b) was performed using a Cox proportional hazards regression model, with hazard ratios (HRs), 95% confidence intervals (CIs), and corresponding *p* values reported. Comparisons of cytotoxicity at the indicated time points in panel (c) were performed using unpaired two-tailed Student’s *t*-test. Longitudinal 3D tumor spheroid cytotoxicity data in panel (d) were analyzed using two-way ANOVA followed by multiple-comparison tests. A two-sided *p* < 0.05 was considered statistically significant.

**Figure S2.**

**Figure S2. HPC-Ⅲ is identified as the optimal regimen to rescue NK92 cell cytotoxicity under acute hypoxia.**

**(a)** Schematic illustration of the experimental workflow for WT-, AH- and three different hypoxic preconditioning conditions (HPC-Ⅰ, HPC-Ⅱ, and HPC-Ⅲ)- treated NK92 cells.

**(b)** Viability of NK92 cells following WT, AH, and three different hypoxic preconditioning conditions was assessed by trypan blue exclusion assay (n = 3).

**(c)** Expression of NKG2D on NK92 cells following tumor cell stimulation under different hypoxic preconditioning conditions was analyzed by flow cytometry, with quantification (E:T = 4:1; time = 4 h; n = 3).

**(d)** Cytotoxic activity of NK92 cells across WT, AH, and three distinct HPC pretreated groups against A549-tdTomato-Luciferase, PC-9-tdTomato-Luciferase, and H1299-tdTomato-Luciferase (E:T = 4:1; n = 3).

**(e)** Cytotoxic activity of NK92 cells against PC-9-tdTomato-Luciferase and H1299-tdTomato-Luciferase 3D tumor spheroids, evaluated using the IncuCyte real-time imaging system. Left panel: Representative red fluorescence images at indicated time points; scale bar = 200 μm. Right panel: Corresponding quantitative analysis (E:T = 8:1; n = 3). HPC in this and all subsequent figures refers to the optimized HPC-III conditioning protocol.

**(f)** Cytotoxicity of WT, AH, and HPC treated NK92 cells against A549-tdTomato-Luciferase, PC-9-tdtomato-Luciferase, and H1299-tdtomato-Luciferase was assessed by luciferase luminescence imaging. Blue and red regions represent the areas of adherent viable cells (E:T = 4:1;time = 24 h; n = 3).

Data are presented as mean ± SD. Statistical analyses were performed using IBM SPSS Statistics, except for the 3D tumor spheroid cytotoxicity assay in panel (e), which was analyzed using GraphPad Prism. Comparisons among WT, AH, HPC-I, HPC-II, and HPC-III groups in panel (b) were analyzed using one-way ANOVA followed by multiple-comparison tests. Comparisons of cytotoxicity at the indicated time points in panel (d) were performed using unpaired two-tailed Student’s *t*-test. Longitudinal 3D tumor spheroid cytotoxicity data in panel (e) were analyzed using two-way ANOVA followed by multiple-comparison tests. A two-sided *p* < 0.05 was considered statistically significant. n.s, *p*>0.05, not significant.

**Figure S3.**

**Figure S3. Transcriptional recovery of NK92 cells after HPC treatment.**

**(a)** The Venn diagram shows the intersection of differentially expressed genes in the three comparisons of AH vs. WT, HPC vs. AH, and HPC vs. WT (n = 3).

**(b)** The left heatmap shows the transcriptional expression levels of NK cell chemotaxis-, cytotoxicity-, and exhaustion-related genes in the WT, AH, and HPC groups; the middle heatmap shows expression changes of glycolysis‒, HIF-1α pathway‒, and oxidative phosphorylation‒related metabolic genes; and the right heatmap displays genes involved in antioxidant and redox regulation. (n = 3).

**Figure S4.**

**Figure S4. MAPK signaling regulates mitochondrial ROS accumulation and biogenesis-associated transcription in NK92 cells.**

**(a)** Correlation analyses in NK cells across individual samples from the GSE131907 dataset. Top left: Correlation between mean MAPK signaling score and mean NKG7 expression score. Top right: Quadratic regression analysis of MAPK signaling score and NKG7 expression score. Bottom left: Correlation between mean hypoxia/HIF1A signature score and mean NKG7 expression score. Bottom right: Correlation between mean hypoxia/HIF1A signature score and mean MAPK signaling score. Each dot represents one sample. Spearman rank correlation analysis was performed for the top-left, bottom-left, and bottom-right panels, and quadratic regression was performed for the top-right panel.

**(b)** Gene set enrichment analysis was performed on the core MAPK cascade components—p38, JNK, and ERK—and their canonical downstream targets; ridge plots illustrate the shift in expression distribution across conditions (n = 3).

**(c)** Western blot analysis of total ERK and phospho-ERK in NK92 cells exposed to 20%, 5%, 3%, or 1% O_2_ (n = 3).

**(d)** Western blot analysis of total and phosphorylated ERK, JNK, and p38 in WT, acute hypoxia (AH), and hypoxic preconditioning (HPC)-treated NK92 cells (n = 3).

**(e)** NK92 cells were treated with 2 μM Anisomycin for 30 min under normoxic conditions, followed by western blot analysis of MAPK-related signaling pathways changes (n = 3).

**(f)** ROS levels in WT and Anisomycin-treated NK92 cells (2 μM, 30 min) were measured under normoxic conditions (n = 3).

**(g)** Intracellular ROS levels were assessed by flow cytometry in WT-, AH-, HPC-, and HPC plus anisomycin-treated (2 μM 30 min, 1% O_2_ 36 h) NK92 cells. Left, representative flow-cytometry histograms; right, corresponding quantification of ROS-positive cells (n = 3).

**(h)** Representative flow-cytometry plots showing intracellular granzyme B (GZMB) expression in WT-, HPC-, and HPC plus Anisomycin-treated NK92 cells (E:T=4:1, n = 3).

**(i)** NK92 cells were treated with PX-478 (20 μM) for 48 h under hypoxic conditions, and changes in the HIF-1α pathway were assessed by western blotting (n = 3).

**(j)** Mitochondrial ROS levels in WT, AH, AH + PX-478, and AH + PX-478 + MAPKi NK92 cells. PX-478 and MAPKi were used at 20 μM and 5 μM, respectively (n = 3).

**(k)** Effects of MitoTEMPO on ROS levels and p38 phosphorylation in NK92 cells under acute hypoxia. Left: Representative flow cytometry histograms showing intracellular ROS levels. Right: Representative Western blot images showing p38 and p-p38 protein levels (n = 3).

Data are presented as mean ± SD where applicable. Correlation analysis in panel (a) was performed using Spearman’s rank correlation test. Statistical significance in panel (f) was determined using an unpaired two-tailed Student’s *t*-test. Comparisons among multiple groups in panel (g) and (j) were analyzed using one-way ANOVA followed by multiple-comparison tests. A two-sided *p* < 0.05 was considered statistically significant. n.s, *p*>0.05, not significant.

**Figure S5.**

**Figure S5. MAPK attenuation promotes PGC-1α-associated mitochondrial remodeling in NK cells in vitro and in vivo.**

**(a)** Representative gating strategy for isolation of peripheral blood NK cells from BALB/c mice. Lymphocytes were identified based on FSC/SSC characteristics, followed by singlet and live-cell gating, and CD49b^+^ cells were subsequently isolated by fluorescence-activated cell sorting.

**(b)** NK92 cells were cultured under normoxic conditions and divided into WT and MAPKi groups. The MAPKi group was treated with 5 μM MAPK inhibitor for 48 h; cells from both groups were then collected for bulk RNA-seq analysis (n=3).

**(c)** Bar plot showing the fold changes of upstream regulators of mitochondrial biogenesis in MAPKi-treated NK92 cells relative to WT cells. The x-axis represents the Log_2_ fold change (n = 3).

**(d)** Bar plot showing the fold changes in mitochondrial gene expression in MAPKi-treated cells compared with WT cells. The x-axis represents Log_2_ fold change (n = 3).

**(e)** Relative *PPARGC1A* mRNA expression in WT and MAPKi-treated NK92 cells was measured by quantitative real-time PCR after 48 h of sesamolin treatment (5 μM) (n = 3).

**(f)** Knockdown efficiency of *PPARGC1A* in NK92 cells transfected with siPPARGC1A was determined by quantitative real-time PCR 48 h after transfection (n = 3). Expression levels were normalized to the negative-control siRNA (NC) group.

**(g)** Oxygen consumption rate (OCR) was measured in NC, MAPKi-treated, and siPPARGC1A plus MAPKi-treated NK92 cells using a Seahorse XF analyzer to assess mitochondrial oxidative phosphorylation. Cells were sequentially exposed to oligomycin, FCCP, and rotenone/antimycin A during the assay. The siPPARGC1A transfection and sesamolin treatment were initiated simultaneously and maintained for 48 h before extracellular flux analysis. (n = 4).

**(h)** Quantitative analysis of basal respiration, maximal respiration, spare respiratory capacity, and ATP production derived from the OCR profiles shown in panel (g) (n = 4).

Data are presented as mean ± SD. Statistical significance between two groups in panels (e) and (f) was determined using an unpaired two-tailed Student’s *t*-test. Comparisons among the three groups in panel (h) were analyzed using one-way ANOVA followed by multiple-comparison tests. n.s, *p*>0.05, not significant.

**Figure S6.**

**Figure S6. MAPK inhibition enhances NK92 cells cytotoxicity under hypoxic stress across multiple models.**

**(a)** Cytotoxic activity of WT-, AH-, and i+AH-treated NK92 cells against PC-9-tdTomato-Luciferase and H1299-tdTomato-Luciferase, evaluated using the IncuCyte real-time imaging system (E:T = 4:1; n = 3).

**(b)** Cytotoxic activity of WT-, AH-, and i+AH-treated NK92 cells against PC-9-tdTomato-Luciferase and H1299-tdTomato-Luciferase 3D tumor spheroids, evaluated using the IncuCyte real-time imaging system; scale bar = 200 μm (E:T = 8:1; n = 3).

**(c)** Cytotoxicity of WT-, AH-, and i+AH-treated NK92 cells against A549-tdTomato-Luciferase, PC-9-tdTomato-Luciferase, and H1299-tdTomato-Luciferase was assessed by luciferase luminescence imaging. Blue and red regions represent areas of adherent viable cells (E:T = 4:1; time = 24 h; n = 3).

**(d)** Representative confocal images of live NK92 cells. Mitochondrial morphology and dynamic architecture were visualized using MitoTracker Deep Red (red fluorescence), with nuclei counterstained with Hoechst (blue fluorescence); scale bar = 2 μm (n = 10).

**(e)** Mitochondrial reactive oxygen species (mtROS) levels in NK92 cells following i+AH treatment were quantified by flow cytometry using MitoSOX Red, with data expressed as the percentage of mtROS-positive cells (n = 3).

**(f)** Intracellular ATP concentration was measured using a luciferase-based luminescent assay to evaluate cellular bioenergetic status (n = 3).

**(g)** Intracellular ROS levels were assessed by flow cytometry in NK92 cells following PPARGC1A knockdown. Cells were transfected with siPPARGC1A and treated with sesamolin as indicated. Twenty-four hours after transfection, cells were exposed to acute hypoxia (1% O_2_) for an additional 24 h. Representative histograms are shown for NC, NC+AH, i+AH, and siPPARGC1A+i+AH groups.

Data are presented as mean ± SD. Statistical analyses were performed using IBM SPSS Statistics, except for the longitudinal spheroid cytotoxicity analysis in panel (b), which was performed using GraphPad Prism. Pairwise comparisons between two independent groups in panel (a) were performed using unpaired two-tailed Student’s *t*-test. Comparisons among three or more groups in panel (e) and panel (f) were analyzed using one-way ANOVA followed by multiple-comparison tests. Longitudinal cytotoxicity data in panel (b) were analyzed using two-way ANOVA followed by multiple-comparison tests. n.s, *p*>0.05, not significant.

**Figure S7.**

**Figure S7. Transcriptomic reversal of hypoxia-induced dysfunction by MAPK inhibition.**

**(a)** Schematic overview of the RNA-seq experimental design for the WT, AH, and i+AH groups. NK92 cells in the i+AH group were pretreated with MAPKi under normoxic conditions and then subjected to acute hypoxia in parallel with the AH group, followed by bulk RNA-seq analysis (n = 3).

**(b)** Venn diagrams depict the overlapping sets of significantly upregulated genes (left) and downregulated genes (right) across three pairwise comparisons: AH vs. WT, i+AH vs. WT, and i+AH vs. AH (n = 3).

**(c)** The left heatmap illustrates transcript-level expression profiles of NK cell–associated functional genes—including chemotaxis-, cytotoxicity-, and exhaustion-related markers—across the WT, AH, and i+AH groups; the right heatmap presents differential expression of metabolic pathway genes involved in glycolysis, HIF-1α signaling, and oxidative phosphorylation (n = 3).

**(d)** Knockdown efficiency of *MAPK8* and *MAPK14* in NK92 cells following combined siRNA-mediated silencing was determined by quantitative real-time PCR 48 h after transfection. Relative gene expression was normalized to the corresponding negative-control (NC) group (n = 3).

**(e)** Intracellular reactive oxygen species (ROS) levels were assessed by flow cytometry in NK92 cells transfected with negative-control siRNA (NC) or combined *MAPK8/MAPK14* siRNAs. Twenty-four hours after transfection, the indicated groups were exposed to acute hypoxia (AH; 1% O_2_) for an additional 24 h. MAPKi-pretreated cells exposed to AH (i+AH) were included as a pharmacological reference. Left, representative flow-cytometry histograms; right, corresponding quantification of ROS-positive cells (n = 3).

**(f)** Oxygen consumption rate (OCR) was measured in NC, NC+AH, and siMAPK+AH NK92 cells using a Seahorse XF analyzer to assess mitochondrial oxidative phosphorylation. Twenty-four hours after siRNA transfection, the indicated cells were exposed to 1% O_2_ for an additional 24 h before extracellular flux analysis. Cells were sequentially treated with oligomycin, FCCP, and rotenone/antimycin A (Rot/AA) during the assay (n = 4).

**(g)** Quantitative analysis of basal respiration, maximal respiration, spare respiratory capacity, and ATP production derived from the OCR profiles shown in panel (f) (n = 4).

**(h)** Schematic illustration of the experimental design (upper left). Healthy or A549 tumor-bearing NCG mice were intravenously injected with untreated NK92 cells (WT) or NK92 cells pretreated with a MAPK inhibitor (MAPKi; 5 μM) for 48 h, as indicated. Peripheral blood was collected 48 h after injection. Representative flow cytometry plots (right) and corresponding quantification (lower left) showing the percentages of CD56^+^ cells and p-p38^+^ cells within the CD56^+^ population(n = 3 mice per group).

Data are presented as mean ± SD. Statistical significance between two groups in panel (d) was determined using an unpaired two-tailed Student’s *t*-test. Comparisons among multiple groups in panels (e), (g), and (h) were analyzed using one-way ANOVA followed by multiple-comparison tests. n.s, *p*>0.05, not significant.

**Figure S8.**

**Figure S8.** **MAPK inhibition enhances iNK cell cytotoxicity under hypoxic stress across multiple models.**

**(a)** Flow cytometry plots showing expression of CD34 and CD45 on day 12 of hiPSC differentiation (n=3).

**(b)** Flow cytometry gating strategy used for the iNK cell analysis shown in Figure. 7b. It successively shows the selection of lymphocytes, exclusion of adherent cells, selection of the CD45^+^CD3^-^ population, and the identification of NK cell subsets based on CD56/CD16 expression (n=3).

**(c)** Comparative analysis of functional marker molecules in hiPSC-derived iNK cells versus NK92 cells (n = 3).

**(d)** Cytotoxic activity of WT-, AH-, and i+AH-treated iNK cells against PC-9-tdTomato-Luciferase and H1299-tdTomato-Luciferase, evaluated using the IncuCyte real-time imaging system (E:T = 4:1; n = 3).

**(e)** Cytotoxic activity of WT-, AH-, and i+AH-treated iNK cells against PC-9-tdTomato-Luciferase and H1299-tdTomato-Luciferase 3D tumor spheroids, evaluated using the IncuCyte real-time imaging system; scale bar = 200 μm (E:T = 8:1; n = 3).

**(f)** Cytotoxicity of WT, AH, and i+AH treated iNK cells against A549-tdTomato-Luciferase, PC-9-tdTomato-Luciferase, and H1299-tdTomato-Luciferase was assessed by luciferase luminescence imaging. Blue and red regions represent the areas of adherent viable cells (E:T = 4:1; time = 24 h; n = 3).

**(g)** Western blot analysis of HIF-1α protein expression in iNK cells (n = 3).

Data are presented as mean ± SD. Statistical analyses were performed using IBM SPSS Statistics, except for panel (e), which was analyzed using GraphPad Prism. Pairwise comparisons between two independent groups in panel (d) were performed using unpaired two-tailed Student’s *t*-test. The analysis in panel (e) was performed using two-way ANOVA followed by multiple-comparison tests. For panel (g), comparisons among three groups were performed using one-way ANOVA followed by multiple-comparison testing. n.s, *p*>0.05, not significant.

**Figure S9.**

**Figure S9. Evaluation of NF-κB signaling and mitochondrial remodeling following MAPK attenuation.**

**(a)** Western blot analysis of NF-κB, phospho-NF-κB, p38, and phospho-p38 in WT and MAPKi-treated iNK cells (n = 3).

**(b)** Left, western blot analysis of NF-κB, phospho-NF-κB, ERK, and phospho-ERK in WT and MAPKi-treated NK92 cells. Right, representative flow-cytometry histogram showing NKG2A expression in WT and MAPKi-treated NK92 cells (n = 3).

**(c)** Western blot analysis validating pharmacological inhibition of NF-κB signaling in iNK cells. Cells were treated with the NF-κB pathway inhibitor BMS-345541 (0.5 μM) for 48 h, followed by western blot analysis of total NF-κB and phospho-NF-κB (n = 3).

**(d)** Representative transmission electron microscopy images and quantification of mitochondrial number per cell in WT, MAPKi-treated, and MAPKi plus BMS-345541-treated iNK cells. MAPKi-treated cells were exposed to sesamolin (5 μM) for 48 h, whereas cells in the combination group were treated simultaneously with sesamolin (5 μM) and BMS-345541 (0.5 μM) for 48 h. Red arrowheads indicate mitochondria; scale bar = 2 μm (n = 20).

Data are presented as mean ± SD. For panel (d), comparisons among three groups were performed using one-way ANOVA followed by multiple-comparison tests. n.s, *p*>0.05, not significant.

**Figure S10.**

**Figure S10. MAPK attenuation enhances in vivo antitumor efficacy and persistence of iNK cells.**

**(a)** Schematic illustration of the in vivo experimental workflow. A549-Luc cells (5 × 10^6^ per mouse) were implanted subcutaneously to establish a human tumor xenograft model. On day 7 post-implantation, mice received intravenous administrations of PBS or 2 × 10^6^ iNK cells (WT or MAPKi-pretreated). iNK cells were administered for a total of three injections on days 7, 13, and 21. No exogenous IL-2 was administered in the iNK model. Tumor progression was monitored by bioluminescence imaging and caliper measurements.

**(b)** Representative longitudinal in vivo bioluminescence images of tumor burden in PBS-, WT iNK-, and MAPKi-iNK-treated mice (PBS: n = 3; WT/MAPKi: n = 4 per group).

**(c)** Longitudinal body weight monitoring of healthy control and tumor-bearing mice receiving PBS, WT iNK cells, or MAPKi-pretreated iNK cells (PBS: n = 3; WT/MAPKi: n = 4 per group).

**(d)** Tumor growth kinetics measured by caliper every 2 days (PBS: n = 3; WT/MAPKi: n = 4 per group).

**(e)** Individual tumor growth trajectories of mice in the PBS, WT iNK, and MAPKi-iNK groups (PBS: n = 3; WT/MAPKi: n = 4 per group).

**(f)** Schematic illustration of the experimental workflow for iNK-cell persistence analysis. Peripheral blood and tumor tissues were collected at the indicated time points after the final iNK infusion and analyzed by flow cytometry to quantify human CD45^+^CD56^+^ iNK cells.

**(g)** Quantification and representative flow cytometry plots showing iNK-cell frequencies in peripheral blood and tumor tissues at 7 and 14 days after the final iNK infusion (WT/MAPKi: n = 3 per group).

Data are presented as mean ± SD. Longitudinal tumor growth and body-weight changes were analyzed using two-way ANOVA followed by multiple-comparison. For iNK persistence analyses, comparisons between WT and MAPKi groups at each indicated time point were performed using an unpaired two-tailed Student’s *t*-test. A two-sided *p* < 0.05 was considered statistically significant. ns, *p*>0.05, not significant.

**Table. S1 Antibody resources and application conditions**

**Primary antibodies**

| **Antigen** | **Reactivity** | **Cat.No.** | | **RRID** | | **Company** | | **Dilution** | |
| --- | --- | --- | --- | --- | --- | --- | --- | --- | --- |
| β-Actin | Mouse | 66009-1-Ig | | AB_2687938 | | Proteintech | | 1:1000 (WB) | |
| p38 MAPK | Rabbit | 8690T | | AB_10999090 | | Cell Signaling  Technology | | 1:1000 (WB) | |
| SAPK/JNK | Rabbit | 9252T | | AB_2250373 | | Cell Signaling  Technology | | 1:2000 (WB) | |
| phospho-p38 (Thr180/Tyr182) | Rabbit | 4511T | | AB_2139682 | | Cell Signaling  Technology | | 1:1000 (WB) | |
| phospho-SAPK/JNK (Thr183/Tyr185) | Rabbit | 4668T | | AB_823588 | | Cell Signaling  Technology | | 1:1000 (WB) | |
| HIF-1α | Rabbit | F0101 | | N/A | | Selleck | | 1:1000 (WB) | |
| NF-kappaB p65 | Rabbit | 8242T | | AB_10859369 | | Cell Signaling  Technology | | 1:1000 (WB) | |
| phospho-NF-kappaB p65 (Ser536) | Rabbit | 3033T | | AB_10859369 | | Cell Signaling  Technology | | 1:1000 (WB) | |
| p44/42 MAPK (Erk1/2) | Rabbit | 4695 | | AB_390779 | | Cell Signaling  Technology | | 1:1000 (WB) | |
| Phospho-p44/42 MAPK (Erk1/2) (Thr202/Tyr204) | Rabbit | 4370T | | AB_2315112 | | Cell Signaling  Technology | | 1:1000 (WB) | |
| **Secondary antibodies** | | | | | | | | | |
| **Product** | | | **Cat.No.** | | **RRID** | | **Company** | | **Dilution** |
| HRP-conjugated Affinipure Goat  Anti-Mouse IgG (H+L) | | | SA00001-1 | | AB_2722565 | | Proteintech | | 1:4000 (WB) |
| HRP-conjugated Affinipure Goat  anti-Rabbit lgG (H+L) | | | SA00001-2 | | AB_2722564 | | Proteintech | | 1:4000 (WB) |

| **Flow Cytometry antibodies** | | | | |
| --- | --- | --- | --- | --- |
| **Product** | **Cat.No.** | **RRID** | **Company** | **Dilution** |
| PE-conjugated anti-human CD107a  Human | 328608 | AB_1186040 | BioLegend | 1:100 |
| APC-conjugated anti-human FasL  Human | 306422 | AB_2922541 | BioLegend | 1:100 |
| PE-conjugated anti-human CD69 | 310906 | AB_314841 | BioLegend | 1:100 |
| PE-conjugated anti-human NKG2D | 320806 | AB_492960 | BioLegend | 1:100 |
| APC-conjugated anti-human NKG2A | 375108 | AB_2888862 | BioLegend | 1:100 |
| BV785-conjugated anti-human NKG2A | 375137 | AB_3068113 | BioLegend | 1:100 |
| APC-conjugated anti-human NKp46 | 331918 | AB_2561650 | BioLegend | 1:100 |
| BV421-conjugated anti-human TRAIL | 308221 | N/A | BioLegend | 1:100 |
| APC-conjugated anti-human TRAIL | 308210 | AB_2564398 | BioLegend | 1:100 |
| PE-conjugated anti-human CXCR6 | 356003 | AB_2561739 | BioLegend | 1:100 |
| PerCP/Cy5.5-conjugated anti-human T-bet | 365907 | N/A | BioLegend | 1:100 |
| PE-conjugated anti-human T-bet | 365903 | AB_2922579 | BioLegend | 1:100 |
| PE-conjugated anti-human CD56 | 304606 | AB_314448 | BioLegend | 1:100 |
| APC-conjugated anti-human CD45 | H0093 | N/A | Selleck | 1:100 |
| PE-conjugated anti-mouse CD49b | 108907 | AB_313414 | BioLegend | 1:100 |
| APC-conjugated anti-mouse CD107a | 121613 | AB_2134486 | BioLegend | 1:100 |
| APC/Cy7-conjugated anti-mouse NKp46 | 137645 | N/A | BioLegend | 1:100 |
| APC-conjugated anti-human CD25 | 302609 | AB_314279 | BioLegend | 1:100 |
| PE-conjugated anti-human CD122 | 339005 | AB_1501056 | BioLegend | 1:100 |
| PE-conjugated anti-human CD132 | 338605 | AB_2233554 | BioLegend | 1:100 |
| BV421-conjugated Mouse IgG1, κ isotype control | 400157 | AB_10897939 | BioLegend | 1:100 |
| PE-conjugated Mouse IgG1, κ isotype control | 400111 | AB_2847829 | BioLegend | 1:100 |
| PerCP/Cy5.5-conjugated Mouse IgG1, κ isotype control | 400149 | AB_893680 | BioLegend | 1:100 |
| APC-conjugated Mouse IgG1, κ isotype control | 400120 | AB_2888687 | BioLegend | 1:100 |
| PE/Cy7-conjugated anti-human CD45 | 368532 | AB_2715892 | BioLegend | 1:100 |
| DAPI | 564907 | AB_2869624 | BD Biosciences | 1:10000 |
| Alexa Fluor® 488-conjugated anti-p38 MAPK Phospho (Thr180/Tyr182) | 690255 | AB_3662338 | BioLegend | 1:100 |
| PE-conjugated anti-human CD34 | 343506 | AB_1731862 | BioLegend | 1:100 |
| BV421-conjugated anti-human CD45 | 563879 | AB_2744402 | BD Biosciences | 1:100 |
| APC-conjugated anti-human CD16 | 302012 | AB_314212 | BioLegend | 1:100 |
| FITC-conjugated anti-human CD3 | 555339 | AB_395745 | BD Biosciences | 1:100 |
| APC-conjugated anti-human/mouse GZMB | 372203 | AB_2687027 | BioLegend | 1:100 |

**Table. S2 Oligonucleotide sequences for RNA interference and quantitative real-time PCR**

**siRNA for RNA interference**

| **Target gene** | **Sense sequence (5′–3′)** | **Antisense sequence (5′–3′)** |
| --- | --- | --- |
| PPARGC1A | GACUAUUGCCAGUCAAUUA | UAAUUGACUGGCAAUAGUC |
| MAPK8 | GUCGAGUAGUGUUAAGAAU | AUUCUUAACACUACUCGAC |
| MAPK14 | GGUCUCUGGAGGAAUUCAA | UUGAAUUCCUCCAGAGACC |

**Primer for quantitative real-time PCR**

| **Target gene** | **Forward primer (5′–3′)** | **Reverse primer (5′–3′)** |
| --- | --- | --- |
| PPARGC1A | TCTGAGTCTGTATGGAGTGACAT | CCAAGTCGTTCACATCTAGTTCA |
| MAPK8 | TCTGGTATGATCCTTCTGAAGCA | TCCTCCAAGTCCATAACTTCCTT |
| MAPK14 | CCCGAGCGTTACCAGAACC | TCGCATGAATGATGGACTGAAAT |
| β-Actin | CATGTACGTTGCTATCCAGGC | CTCCTTAATGTCACGCACGAT |
